# The Genome of *Nectria haematococca*: Contribution of Supernumerary Chromosomes to Gene Expansion

**DOI:** 10.1371/journal.pgen.1000618

**Published:** 2009-08-28

**Authors:** Jeffrey J. Coleman, Steve D. Rounsley, Marianela Rodriguez-Carres, Alan Kuo, Catherine C. Wasmann, Jane Grimwood, Jeremy Schmutz, Masatoki Taga, Gerard J. White, Shiguo Zhou, David C. Schwartz, Michael Freitag, Li-jun Ma, Etienne G. J. Danchin, Bernard Henrissat, Pedro M. Coutinho, David R. Nelson, Dave Straney, Carolyn A. Napoli, Bridget M. Barker, Michael Gribskov, Martijn Rep, Scott Kroken, István Molnár, Christopher Rensing, John C. Kennell, Jorge Zamora, Mark L. Farman, Eric U. Selker, Asaf Salamov, Harris Shapiro, Jasmyn Pangilinan, Erika Lindquist, Casey Lamers, Igor V. Grigoriev, David M. Geiser, Sarah F. Covert, Esteban Temporini, Hans D. VanEtten

**Affiliations:** 1 Department of Plant Sciences, University of Arizona, Tucson, Arizona, United States of America; 2 Massachusetts General Hospital, Boston, Massachusetts, United States of America; 3 BIO5 Institute and Department of Plant Sciences, University of Arizona, Tucson, Arizona, United States of America; 4 Department of Biology, Duke University, Durham, North Carolina, United States of America; 5 United States Department of Energy Joint Genome Institute, Walnut Creek, California, United States of America; 6 Joint Genome Institute—Stanford Human Genome Center, Palo Alto, California, United States of America; 7 Hudson Alpha Genome Sequencing Center, Hudson Alpha Institute for Biotechnology, Huntsville, Alabama, United States of America; 8 Department of Biology, Okayama University, Okayama, Japan; 9 Laboratory for Molecule and Computational Genomics, University of Wisconsin, Madison, Wisconsin, United States of America; 10 Department of Biochemistry and Biophysics and Center for Genome Research and Biocomputing, Oregon State University, Corvallis, Oregon, United States of America; 11 The Broad Institute, Cambridge, Massachusetts, United States of America; 12 Architecture et Fonction des Macromolécules Biologiques, CNRS, Universités Aix-Marseille I & II, Marseille, France; 13 Institut National de la Recherche Agronomique, Centre de recherche de Sophia-Antipolis, Sophia-Antipolis, France; 14 Department of Molecular Sciences, University of Tennessee, Memphis, Tennessee, United States of America; 15 Department of Cell Biology and Molecular Genetics, University of Maryland, College Park, Maryland, United States of America; 16 Department of Biological Sciences, Purdue University, West Lafayette, Indiana, United States of America; 17 Plant Pathology, University of Amsterdam, Amsterdam, The Netherlands; 18 South West Center for Natural Products Research and Commercialization, Office of Arid Lands Studies, University of Arizona, Tucson, Arizona, United States of America; 19 Department of Soil, Water, and Environmental Science, University of Arizona, Tucson, Arizona, United States of America; 20 Department of Biology, Saint Louis University, St. Louis, Missouri, United States of America; 21 Department of Plant Pathology, University of Kentucky, Lexington, Kentucky, United States of America; 22 Institute of Molecular Biology, University of Oregon, Eugene, Oregon, United States of America; 23 Fusarium Research Center, Department of Plant Pathology, The Pennsylvania State University, University Park, Pennsylvania, United States of America; 24 Warnell School of Forestry and Natural Resources, University of Georgia, Athens, Georgia, United States of America; 25 Vilmorin Inc., Tucson, Arizona, United States of America; University of California San Francisco, United States of America

## Abstract

The ascomycetous fungus *Nectria haematococca*, (asexual name *Fusarium solani*), is a member of a group of >50 species known as the “*Fusarium solani* species complex”. Members of this complex have diverse biological properties including the ability to cause disease on >100 genera of plants and opportunistic infections in humans. The current research analyzed the most extensively studied member of this complex, *N. haematococca* mating population VI (MPVI). Several genes controlling the ability of individual isolates of this species to colonize specific habitats are located on supernumerary chromosomes. Optical mapping revealed that the sequenced isolate has 17 chromosomes ranging from 530 kb to 6.52 Mb and that the physical size of the genome, 54.43 Mb, and the number of predicted genes, 15,707, are among the largest reported for ascomycetes. Two classes of genes have contributed to gene expansion: specific genes that are not found in other fungi including its closest sequenced relative, *Fusarium graminearum*; and genes that commonly occur as single copies in other fungi but are present as multiple copies in *N. haematococca* MPVI. Some of these additional genes appear to have resulted from gene duplication events, while others may have been acquired through horizontal gene transfer. The supernumerary nature of three chromosomes, 14, 15, and 17, was confirmed by their absence in pulsed field gel electrophoresis experiments of some isolates and by demonstrating that these isolates lacked chromosome-specific sequences found on the ends of these chromosomes. These supernumerary chromosomes contain more repeat sequences, are enriched in unique and duplicated genes, and have a lower G+C content in comparison to the other chromosomes. Although the origin(s) of the extra genes and the supernumerary chromosomes is not known, the gene expansion and its large genome size are consistent with this species' diverse range of habitats. Furthermore, the presence of unique genes on supernumerary chromosomes might account for individual isolates having different environmental niches.

## Author Summary

*Nectria haematococca* MPVI occurs as a saprophyte in diverse habitats and as a plant and animal pathogen. It also was the first fungus shown to contain supernumerary chromosomes with unique habitat-defining genes. The current study reveals that it has one of the largest fungal genomes (15,707 genes), which may be related to its habitat diversity, and describes two additional supernumerary chromosomes. Two classes of genes were identified that have contributed to gene expansion: 1) specific genes that are not found in other fungi, and 2) genes that are present as multiple copies in *N. haematococca* but commonly occur as a single copy in other fungi. Some of these genes have properties suggesting their acquisition by horizontal gene transfer. We show that the three supernumerary chromosomes are different from the normal chromosomes; they contain more repeat sequences, are particularly enriched in unique and duplicated genes, and have a lower G+C content. Additionally, the biochemical functions of genes on these chromosomes suggest they may be involved in niche adaptation. The dispensable nature and possession of habitat-determining genes by these chromosomes make them the biological equivalent of bacterial plasmids. We believe they contribute to microbial diversity and have been overlooked in models of fungal evolution.

## Introduction

The fungus *Nectria haematococca*, commonly referred to by its asexual name *Fusarium solani*, is a member of a monophyletic clade that includes over 50 phylogenetic species known as the “*Fusarium solani* species complex” [1],[2]. Members of the *F. solani* species complex are able to colonize an impressive variety of environments. As saprobes, they are present in agricultural and non-cultivated habitats, such as forests, scrub communities, savannahs, prairies, swamps, littoral zones, coastal zones, and deserts [3]. As pathogens, members of this complex are responsible for disease on ∼100 genera of plants [4], and they represent one of the most important group of pathogens associated with opportunistic fungal infections and keratitis in humans [2], [5]–[8]. Because of their diverse host range, some members have been proposed for the biological control of weeds and other pathogens [9]–[11]. Extreme environments are not beyond the reach of these fungi. *F. solani* is among the fungal species recovered from the highly radioactive inner parts of the damaged nuclear reactor at Chernobyl [12]. These fungi are capable of growing in anaerobic conditions in the soil [13] and are tolerant to many compounds shown to be toxic to other fungi [14],[15]. *F. solani* also has been found growing in the caves at Lascaux, France where it is damaging the 15,000 year-old paintings [16]. The ability of these fungi to adapt to so many different environments reflects their genetic plasticity and metabolic diversity. Individual members of this species complex can degrade hydrocarbons, organofluorine compounds, lignin, metal cyanides, and pesticides in the soil [17]–[29].

The most extensively studied species of the *F. solani* complex is “mating population” (MP) VI of *N. haematococca* (also called *Haematonectria haematococca* [30]). The term “mating population” defines a group of isolates that are sexually fertile with one another, indicating that they are a biological species. Like other *F. solani* species, *N. haematococca*a MPVI isolates can live in many habitats [31] and classical and molecular genetic analyses have demonstrated that the genes controlling the ability of individual isolates to colonize specific habitats are located on conditionally-dispensable supernumerary chromosomes (“CD chromosomes”), which were first described in fungi in 1991 using *N. haematococca* MPVI [32]. CD chromosomes are defined as supernumerary chromosomes that are not required for growth under all conditions but confer an adaptive advantage in certain habitats [33]. Subsequent research has demonstrated that in *N. haematococca* MPVI, genes on these chromosomes are involved in resistance to plant antimicrobials, utilization of specific carbon and nitrogen sources, and in host-specific pathogenicity [33]–[35]. In addition, the properties of the chromosomes and the properties of the genes on these chromosomes, suggest that some of these genes and perhaps even the entire chromosome(s) might have been acquired through horizontal gene transfer (HGT) and have properties similar to the genomic islands of bacteria [36]. The genomic sequence of *N. haematococca* MPVI has not only the potential to reveal a multitude of metabolic pathways involved in inhabiting many different types of environments, but also to expand on our understanding of the impact of gene flow on fungal evolution.

## Results

### General genome features

Optical mapping revealed that *N. haematococca* MPVI isolate 77-13-4 has 17 chromosomes ranging from 530 kb to 6.52 Mb and that the physical size of the genome, 54.43 Mb, is larger than that of any other published ascomycete (Table S1). This is 15% greater than that of the most closely related sequenced fungus, *Fusarium graminearum* (sexual name: *Gibberella zeae*), which is known to have undergone significant gene expansion itself [37]. The average gene length (1.67 kb), number of exons (3.08), intron size (84 nt), and the size of the encoded protein (480 aa) (Table S2) are similar to other sequenced ascomycetes [38],[39]. Of the gene families in *N. haematococca* MPVI with at least ten genes, 77% (226 of 293) have more genes than their counterpart in *F. graminearum*; 18% have more than twice as many genes (Figure S1). As might be expected for a metabolically diverse fungus that can live in so many habitats, among the gene families with the greatest numerical increases are carbohydrate-active enzymes, oxidoreductases, and various monooxygenases and dioxygenases (Table S3).

### Chromosomal location of genes similar to other fungi

To determine why *N. haematococca* MPVI might have more genes than *F. graminearum* and to see if these “extra” genes are similar to genes from other fungi, the proteome of *N. haematococca* MPVI was compared to the genomes of eight other sequenced fungi (*F. graminearum*, *Aspergillus oryzae*, *A. nidulans*, *Coccidioides immitis*, *Chaetomium globosum*, *Magnaporthe oryzae*, *Neurospora crassa*, and *Saccharomyces cerevisiae*). The predicted genes of *N. haematococca* MPVI were classified into three groups: those most similar to *F. graminearum*, those most similar to genes from the other fungi used in the comparison, or those with no similarity to genes from any of the included genomes. 61.5% were most similar to *F. graminearum* genes, 28.5% were more similar to the genes from other fungi, and 6.4% had no good match with any of the other genomes. Among the genes with the highest similarity outside *F. graminearum*, the highest similarity was to *Aspergillus* species (1786 genes), particularly to *A. oryzae* (811 genes).

**Figure 1:**
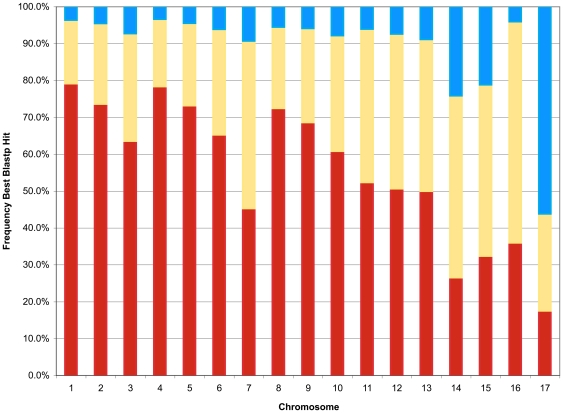
TBLASTN analysis of genes on each chromosome. The relative frequency of the best TBLASTN hits for proteins from each *N. haematococca* MPVI chromosome. The red line depicts hits to the *F. graminearum* genome, the yellow line depicts hits to one of the seven other fungal species, and the blue line represents hits to none of the fungal species included in the search.

The percentage of the genes in each category also was determined for each chromosome. With the exception of chromosome 7, the majority (>60%) of the genes on the large chromosomes (chromosomes 1–10; ranging in size from 6.52 to 3.00 Mb) are highly similar to genes found in *F. graminearum* (Figure 1), suggesting that these chromosomes are largely derived from an ancestor common to both *N. haematococca* MPVI and *F. graminearum*. For chromosomes 7 (3.83 Mb) and 11–13 (2.72–2.19 Mb about half of the genes are more similar to genes of fungal species other than *F. graminearum*. Interestingly, most of the genes on chromosomes 14–16 (1.57–0.56 Mb) are more similar to genes in other fungi than to genes in *F. graminearum*, suggesting that these chromosomes are either enriched for very ancient sequences lost from *F. graminearum*, or the genes were horizontally transferred into *N. haematococca* MPVI from distantly related fungi. In particular, 20.3%, 19.7%, and 41.3% of the genes on chromosomes 14, 15, and 16, respectively, are most similar to sequences from *Aspergillus* species. Chromosome 14 also corresponds to a previously studied CD chromosome that carries a cluster of genes for pea pathogenicity (*PEP* genes) [40]. More than 50% of the proteins encoded by genes on chromosome 17 (530 kb) have no significant similarity to genes from any of the eight fungi selected for comparison (Figure 1) suggesting it is also enriched for very ancient sequences or the genes were derived by horizontal transfer.

### Presence of orthologs, unique genes, duplicated genes, and possible “pseudoparalogs”

**Figure 2:**
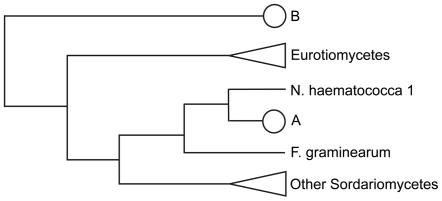
Phylogenetic placement of paralogs in *N. haematococca* MPVI. *N. haematococca 1* is the ortholog. (A) Placement of a gene at this position implies a recent gene duplication. (B) Placement of a gene at this position indicates the gene may be a pseudoparalog.

**Figure 3:**
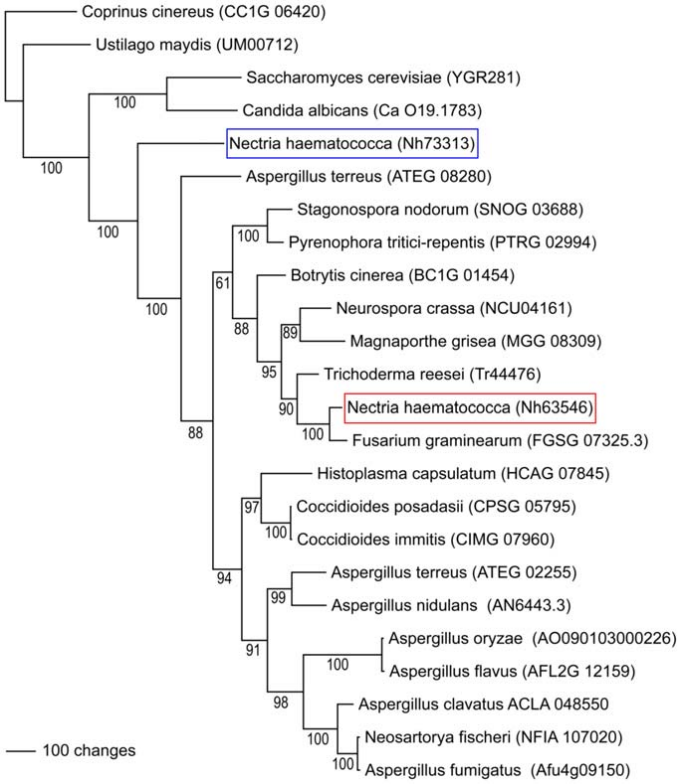
The phylogenetic relationship of the ABC transporter YOR1 from selected fungal genomes. Maximum parsimony analysis was used to establish the phylogenetic relationship between the ortholog (Nh63546, red box) and the pseudoparalog (Nh73313, blue box) of *N. haematococca* MPVI.

Many gene families in *N. haematococca* MPVI are larger than the same families in other ascomycetes. In an effort to investigate further the origin of these additional genes, a phylogenetic analysis was carried out on five gene families (ABC transporters, carbohydrate-active enzymes, P450 monooxygenases, binuclear zinc transcription factors, and chromatin genes; Tables S4, S5, S6, S7, S8). This analysis divided the extra genes into two groups: 1) genes specific to *N. haematococca* MPVI that are not found in *F. graminearum* and other fungi, and 2) genes that are present as multiple copies in *N. haematococca* MPVI but are commonly represented by a single copy in other fungi. In some cases the multiple copies (i.e., paralogs) appear to result from lineage-specific gene duplication (Figure 2). However, in other cases the paralogs are more closely related to a gene from distantly related fungal species (often an *Aspergillus* species); thus, the gene phylogeny does not reflect the species phylogeny (Figure 2). A specific example of this phenomenon is shown in Figure 3 using the phylogenetically conserved ABC transporter gene *YOR1*. The *YOR1* homologs in 27 fungi were identified by a protein similarity search, and their phylogenetic relationship determined (Figure 3). *N. haematococca* MPVI has two copies of *YOR1* (Nh63546 and Nh73313). Nh63546 appears to be an ortholog of *YOR1* in *F. graminearum* (FGSG_07325), which is the closest relative of *N. haematococca* MPVI included in this analysis. In contrast, Nh73313 does not demonstrate the expected phylogenetic placement (Figure 3). While grouped with other *YOR1* homologs, Nh73313 appears distantly related to the *F. graminearum YOR1* and its *N. haematococca* MPVI ortholog, Nh63546. Genes that demonstrate an incongruent phylogenetic topology, as illustrated in Figure 2 and as specifically shown for Nh73313 in Figure 3, have been called ‘pseudoparalogs’ [41]. A pseudoparalog is a copy of a gene that appears paralogous in a single genome analysis, but when sequences from another genome are included, it appears as if the gene were transferred laterally into the genome. However, it has been pointed out recently that the same topology can occur if there is gene duplication, diversification, and differential gene loss (DDL) [42],[43]. Specific and duplicated genes were observed within all five gene families and apparent pseudoparalogous genes were found in all families except the chromatin genes. An example of a pseudoparalog for each gene family is given in the footnotes of Tables S4, S5, S6, S7.

**Figure 4:**
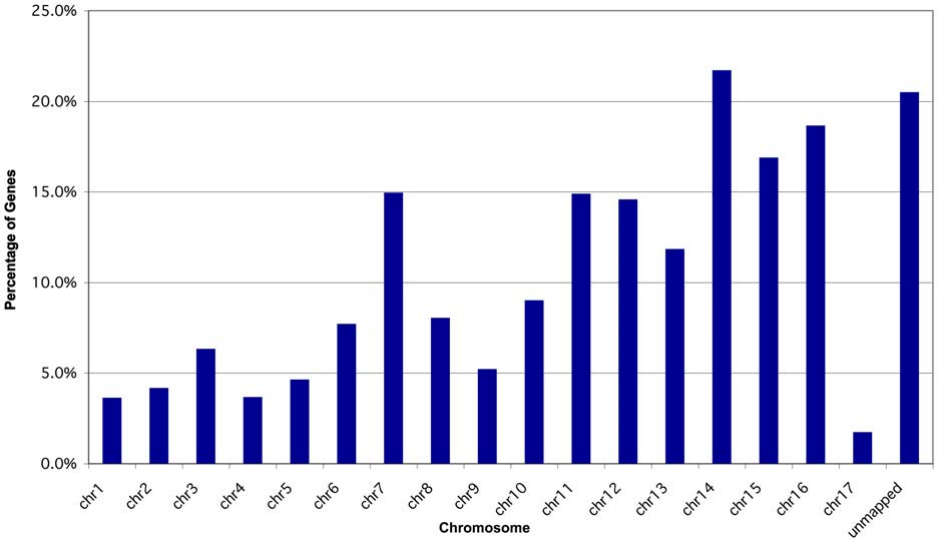
Chromosomal locations of possible pseudoparalogs. The percentage for each chromosome is based on the number of possible pseudoparalogs out of the total number of genes on that chromosome.

Since it has been proposed that HGT could account for some of the genes on the 1.6-Mb CD chromosome of *N. haematococca* MPVI [34],[36], and four of the five expanded gene families included pseudoparalogs, a global analysis of the genome was undertaken to identify possible pseudoparalogs and genes unique to *N. haematococca* MPVI. Reciprocal BLASTp searches between the *F. graminearum* and *N. haematococca* MPVI proteomes resulted in the identification of 8,922 possible orthologs representing 56.8% of the genes in *N. haematococca* MPVI. The remaining 6,785 genes in *N. haematococca* MPVI were identified as ‘unique’ genes. It is within these unique genes that pseudoparalogs are found. To identify possible pseudoparalogs, the unique genes from *N. haematococca* were compared to the *F. graminearum* proteome and the orthologs of *N. haematococca* MPVI with a reciprocal BLASTp approach. A liberal arbitrary cut off of 40% identity over a 40-amino acid length was used to limit the results. A non-stringent cut off for orthologs was used as it created a more comprehensive search for possible pseudoparalogs. Those unique genes that had mutual best hits to both genes of a *F. graminearum-N. haematococca* MPVI ortholog-pair were classified as possible pseudoparalogs. For example, two *CAX* (calcium exchange) transporter genes were found in this set; one (Nh65123) is orthologous to *F. graminearum* FGSG_01606 and the phylogenetic placement of the second, Nh101770, suggests it is a pseudoparalog (Figure S2). Using this approach, 1,331 possible pseudoparalogs were identified (Figure 4). It should be noted that this approach does not differentiate between duplicated and pseudoparalogous genes.

### The G+C percentage and codon usage of orthologs, unique and possible pseudoparalogs in the *N. haematococca* MPVI genome

**Figure 5:**
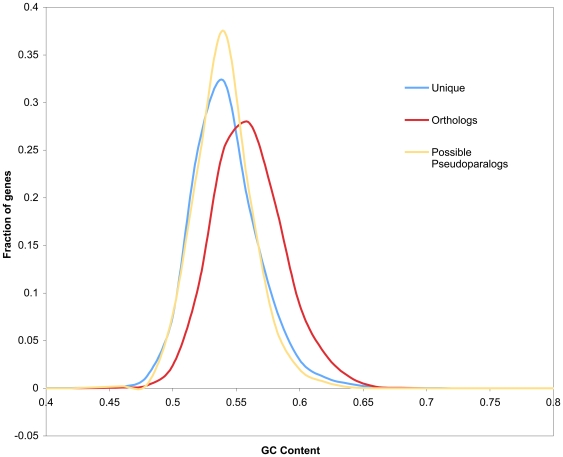
G+C content of orthologs, possible pseudoparalogs, and unique genes.

Outside of the A+T rich repeated regions typically associated with pericentromeric or centromeric regions, the G+C content is generally consistent among genes within a genome [44],[45]. However, sequences introduced into a genome sometimes retain characteristics of the donor genome. This observation has led to the use of G+C content and codon usage to identify regions in prokaryotic genomes that might have arisen via HGT [44],[46],[47]. The large data set of the groups of genes found in *N. haematococca* MPVI allowed an analysis of the G+C content of the orthologs to *F. graminearum*, *N. haematococc*a MPVI unique genes, and possible pseudoparalogs. The overall %G+C content of the orthologs was 55.2% versus 53.3% for the unique genes (P = <2.2×10^−16^) (Figure 5), while the %G+C of the 3^rd^ position of the codon was 61.5% for the orthologs versus 57.8% for the unique gene set (P = <2.2×10^−16^) (data not shown). This same overall %G+C difference was observed when the possible pseudoparalogs were compared to the orthologous genes (Figure 5).

An analysis of the codon usage of the orthologs and the unique genes was used to identify several differences between the two groups (Table 1). To determine the frequency of each codon for an amino acid, the number of times a particular codon occurred was compared to the occurrence of all the codons for that amino acid. Two of the nine codons that appeared at different frequencies in the two sets of genes, GGG and TTA, had been identified previously as having a different frequency of usage in some of the genes on the CD chromosome compared to genes on other chromosomes [48]. The two codons for the amino acid lysine, in particular, exemplify the difference between the two sets of genes. The codon AAA is used 33,924 times in the set of unique genes but only 30,002 times in the set of orthologous genes, even though the set of orthologous genes is ∼50% larger than the set of unique genes. These codon biases also were observed among the pseudoparalogs, although the smaller number of genes did not allow as many comparisons to be made (data not shown).

**Table 1:** Differences in codon usage between orthologs and unique genes in *N. haematococca* MPVI.

		Orthologous gene set		Unique gene set		P-value*
		Frequency	%	Frequency	%	
G	GGG	25856	11.0	30123	16.1	0
	GGT	60388	25.7	42247	22.6	0
I	ATA	13655	8.3	18167	12.9	6.3×10^−250^
	ATT	52813	32.2	45373	32.2	6.3×10^−250^
K	AAG	138188	82.2	86261	71.8	0
	AAA	30002	17.8	33924	28.2	0
L	CTC	101513	33.8	72675	28.9	0
	TTA	7590	2.5	10569	4.2	0
R	CGA	47522	23.0	30574	19.5	0
	AGA	25285	12.3	25746	16.4	0
V	GTA	15791	7.5	16471	9.8	7.5×10^−152^
	GTC	90688	43.2	68607	40.8	7.5×10^−152^

*P-values were determined using Fisher's exact test (2-tailed) to determine the significance of codon usage difference between genes identified as orthologous and those within the unique gene set.

### Supernumerary chromosomes

**Figure 6:**
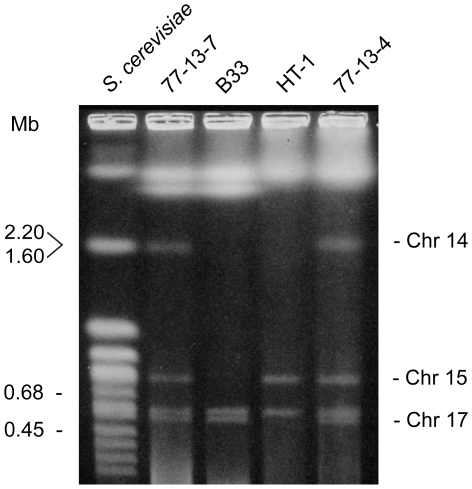
Partial electrophoretic karyotypes of 77-13-7, 77-13-4, and two isolates, B33 and HT-1, derived from 77-13-7 and 77-13-4, respectively. Pulsed-Field Gel Electrophoresis conditions that allowed the resolution of the smaller chromosomes were used.

**Figure 7:**
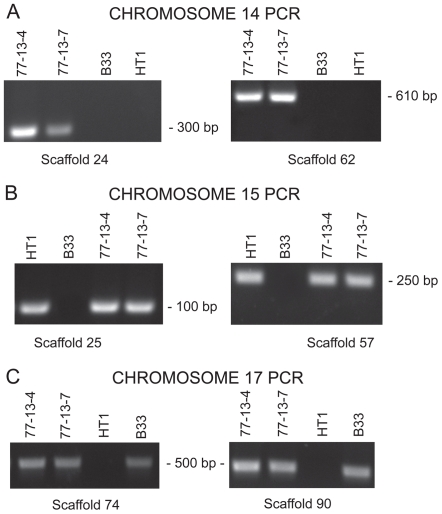
Detection of chromosome-specific sequences found on the ends of chromosomes 14, 15, and 17 in isolates 77-13-7, 77-13-4, and two isolates, B33 and HT-1, derived from 77-13-7 and 77-13-4, respectively. Primers from the scaffolds at the ends of the chromosomes were used to produce PCR products from the end of chromosome 14 (A), chromosome 15 (B), and chromosome 17 (C).

Chromosomes 14–17 are distinctive in their gene content and, as previously mentioned, chromosome 14 is a CD chromosome. In previous studies ([35],[49] and unpublished data) in which isolates were selected for the loss of traits linked to chromosome 14, two isolates (B-33 and HT1) appeared also to have lost another small chromosome. Pulsed field gel electrophoresis experiments revealed that, along with chromosome 14, chromosome 15 appeared to have been lost from B-33 and chromosome 17 appeared to have been lost from HT1 (Figure 6). The loss of all three of these chromosomes was further substantiated by demonstrating that B-33 and HT1 lacked corresponding chromosome-specific sequences on the ends of each assembled chromosome (Figure 7). Therefore, chromosomes 15 and 17, like chromosome 14, are supernumerary chromosomes.

### Repetitive DNA and RIP

**Figure 8:**
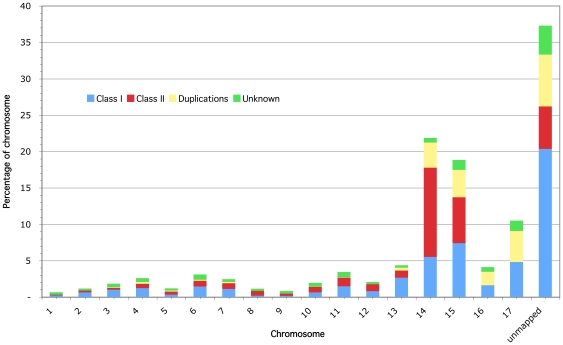
Distribution of repeat elements in the *N. haematococca* genome. The bar graphs show homologs of previously known or novel transposable elements (Class I, retrotransposons; Class II, DNA transposons; Duplications, repeated regions that are mutated duplicated genes, usually with TE fragments; unknown, repeats that do not match any known or hypothetical proteins). A t-test on the log odds ratio of repetitive and unique fractions of each chromosome revealed that chromosomes 14, 15, and 17 had a higher repetitive content than the other chromosomes (p = 0.01416).

Repeated DNA accounts for 5.1% of the *N. haematococca* MPVI genome (Table S9). Over half of the repeated sequences (56.1%) are in the unmapped scaffolds, which are 37.2% repetitive. The mapped repeated sequences are unevenly distributed within the genome with chromosomes 14, 15 and 17 containing 32% of the repetitive DNA despite accounting for only 4% of the mapped genome (Table S9, Figure 8). Chromosome 14 is particularly rich in repeats being 21.8% repetitive DNA. Chromosome 14 also contains a disproportionately large number of the DNA transposons found in *N. haematococca* MPVI (Figure 8).

Interestingly, very few of the repeats in *N. haematococca* MPVI showed a high percentage of identity with each other (Figure S3) suggesting that repeat-induced point mutation (RIP) is potentially involved in the evolution of this genome as it is in *N. crassa* and other ascomycetes [50],[51]. *N. haematococca* MPVI has a homolog of *RID* (RIP defective gene), a putative cytosine methyltransferase that is necessary for RIP [52]. In *N. crassa*, RIP introduces C∶G to T∶A mutation and the degree of RIP can be assessed by calculating TpA/ApT ratios [51]. When this was done for *N. haematococca* MPVI, the ratio suggested that 71.6% of the repetitive sequences but only 3.7% of the unique sequences had been subjected to RIP. Specific analysis of select duplicated genes in *N. haematococca* MPVI also demonstrated the presence of nucleotide changes that are hallmarks of RIP (Figure S4). Finally, RIP was experimentally demonstrated in *N. haematococca* MPVI by analyzing progeny from a cross in which one parent contained a duplicated gene for hygromycin resistance (hygromycin phosphotransferase, *hph*). All progeny were hygromycin sensitive, as would be expected if RIP were operative. When a portion of the *hph* gene from two of the progeny was amplified by PCR, the PCR products had G to A mutations at TpG sites (Figure S5) confirming that RIP is active in *N. haematococca* MPVI. However, PCR products that represented the entire *hph* gene and showed no sign of RIP were also obtained from the same progeny. While RIP can occur in *N. haematococca* MPVI, some additional mechanisms that have been shown to be operative in other fungi, e.g., either ‘methylation induced premeiotically’ (MIP) or the small RNA-dependent ‘quelling’, may be responsible for the silencing of duplicated genes in the absence of point mutations [53],[54]. Indeed, the *masc1* gene, a homolog of *RID* is the sole gene known to be essential for MIP in *Ascobolus immersus* [55] and all genes known to play essential roles in quelling or meiotic silencing by unpaired DNA (“MSUD”) in *N. crassa* (52) have homologs in *N. haematococca* MPVI (data not shown).

### Physical properties of genes on specific chromosomes and G+C content of the chromosomes

The small chromosomes of *N. haematococca* MPVI have several unique properties and these are also observed in the physical properties of their genes (Table S10). The average gene density for the whole genome is 307 genes per Mb (Table S2), but only 223 and 248 genes per Mb for chromosomes 14 and 17, respectively. This may be a reflection of the higher amount of repetitive DNA in these chromosomes. However, the average gene size is also smaller for these chromosomes (1,376 nt for chromosome 14, 1,327 nt for chromosome 15, and 1,484 nt for chromosome 17 versus an average of 1,674 nt for the total genome). The genes on chromosomes 14 and 15 also have fewer exons than the average for the total genome (2.9 versus 3.1) (Tables S2 and S10). In addition, the G+C content (48.2%) of the supernumerary chromosomes is lower than that of the other chromosomes (51.7%) (Table S10).

### Location and number of specific genes of interest

Not all gene families in *N. haematococca* MPVI are exceptionally large. For example, the classes present and number of protein kinases are very similar to *S. cerevisiae* (Table S11). Because of the diverse habitats and broad host range of *N. haematococca* MPVI, it might be expected that it would have large numbers of nonribosomal peptide synthetase (NRPS) and polyketide synthetase (PKS) genes as some of these have been shown to synthesize important virulence factors and to contribute to pathogen diversity [56]–[60]. However, the number of NRPS and PKS genes is actually lower than that found in most fungi (Table S12) and these genes are not on the small chromosomes. Another class of genes that has been implicated in the adaptation to ecological niches is that encoding small, secreted proteins [61],[62]. *N. haematococca* MPVI has 746 of these genes, which is about average for plant pathogens (Table S13).

## Discussion

*N. haematococca* MPVI has a particularly large genome compared to most sequenced ascomycetes. The large number of genes is consistent with its metabolic, ecological, and biological diversity [31],[34]. Among the factors that have contributed to its large size are the supernumeary chromosomes (chromosomes 14, 15, and 17). The mapped portions of these chromosomes contain 418 genes (Table S10). Based on the sizes of these chromosomes as determined by the optical map, 1.5 of 3.5 Mb (approximately 40%) of these chromosomes remains unassigned. One of the unmapped scaffolds contains a gene (*MAK1*) known to be on a 1.6-Mb CD chromosome in another isolate, 156-30-6 [63]. Thus, there are probably substantially more than 418 genes on these chromosomes.

It has been verified experimentally that at least three *N. haematococca* MPVI chromosomes (chromosomes 14, 15, and 17) are dispensable (Figures 6 and 7). These supernumerary chromosomes also have relatively few *F. graminearum* orthologs, but contain unique genes, a disproportionate number of possible pseudoparalogs, a lower G+C content, and a high amount of repetitive DNA with an enrichment of specific types of repeats. Chromosome 14 is a CD chromosome because genes on chromosome 14 increase the habitats available for *N. haematococca* MPVI [34]. Whether chromosomes 15 and 17 also contribute to the ability of this fungus to occupy more niches and are thereby CD chromosomes, is yet to be established. BLAST searches of the genes on chromosomes 14, 15, and 17 revealed similarity to genes involved in a variety of activities, e.g., biofilm formation, utilization of unique nutrients, etc. (data not shown), which are consistent with the involvement of these genes in habitat specialization. Like chromosome 14, the genes on chromosomes 15 and 17 differ in size from those on the other chromosomes (Table S10).

B chromosomes, a well-known type of supernumerary chromosome [64],[65], also have large amounts of repetitive DNA. However, classical B chromosomes are highly heterochromatic, have very few, if any, active genes, and are for the most part transcriptionally inactive [64]–[66]. In contrast to classical B chromosomes, the CD chromosomes of *N. haematococca* MPVI contain functional genes for pathogenicity, antibiotic resistance, and the utilization of unique carbon/nitrogen sources [34]. In addition, based on ESTs [67], about 10% of the genes on the small chromosomes are expressed during growth in defined media, even though the BLAST searches did not detect genes involved in essential core functions (data not shown).

The origin of the supernumerary chromosomes is unknown. B chromosomes are often proposed to be derived from A chromosomes (‘normal’ chromosomes) [64]–[66]. However, restriction patterns used to construct the optical map did not reveal regions of similarity between any of *N. haematococca* MPVI three supernumerary chromosomes and the other chromosomes. Attempts to demonstrate synteny within the *N. haematococca* MPVI genome by identifying three-gene pairs in the same order and orientation failed to demonstrate any large-scale segmental duplications. However, six paired regions of up to 50 kb were found. Although three of these regions are on chromosome 14, two of the regions are similar to DNA on unmapped scaffolds and one is on chromosome 6, which appears to be part of the main genome complement. The RIP system of *N. haematococca* MPVI acting on ancient genome duplications in combination with gene loss might conceal any large-scale duplications, if they occurred [68]. It also has been proposed that B chromosomes could arise from A chromosomes following interspecific hybridization in sexual crosses [66]. In this situation, it is possible that the B chromosome would be the only remnants of an original A chromosome. Extending the mechanism of hybridization to include the well-known parasexual cycle that occurs in some fungi provides another explanation of the origin of supernumerary chromosomes in *N. haematococca* MPVI. As with sexual hybridization there are numerous barriers between vegetative fusion of different fungal species with the major one being vegetative incompatibility [69], which acts at the intraspecies level. However, as recently pointed out by Sanders [70], it would seem unlikely that the barriers to vegetative fusion are so efficient as to prevent entirely the fusion of fungal cells and the subsequent exchange of DNA. Such an event of DNA exchange need not be common; in principle it need happen only once, particularly if the transferred genetic material provided a selective advantage. The exchange of supernumerary chromosomes has been demonstrated in the laboratory, but thus far only between members of the same species [71],[72].

Supernumerary chromosomes do not explain entirely the large size of the *N. haematococca* MPVI genome. Unique genes on non-supernumerary chromosomes with atypical G+C content and codon preference are also potential pseudoparalogs obtained by HGT [41],[44],[46],[47]. Although HGT has not been considered to be a common mechanism of gene acquisition in fungi [73], there is an increasing number of examples of apparent HGT [74]–[76]. However, the data are often inconclusive and subject to different interpretations [77]. For example, genes may be classified as pseudoparalogs because of lineage-specific gene loss, poor phylogenetic taxon sampling, artifacts due to long branch attraction, and other variables that obfuscate gene origins via DDL or HGT [42],[43]. Many of the properties of the supernumerary chromosomes are similar to genomic islands of *A. fumigatus* [42]. These species-specific regions of DNA can be as large as 400 kb, and contain *A. fumigatus*-specific genes and large amounts of repetitive DNA. Genomic islands have smaller genes with fewer exons than the core genes found in *Aspergillus* species. The genes on these genomic islands, like those on the supernumerary chromosomes, encode metabolic processes that appear to be involved in the adaptation to different ecological niches. The origin of the genes on these islands was originally attributed to HGT, but after comparisons to genomes of additional *Aspergillus* species, Fedorova et al. [42] proposed that the genes on the genomic islands of *Aspergillus* arose by DDL. Nevertheless, the ability of *N. haematococca* MPVI to acquire genes by HGT or a similar mechanism would bypass the evolutionary restraint that an active RIP system places on a fungus by hindering the utility of gene duplications as a means to create new genes functions [50].

**Figure 9:**
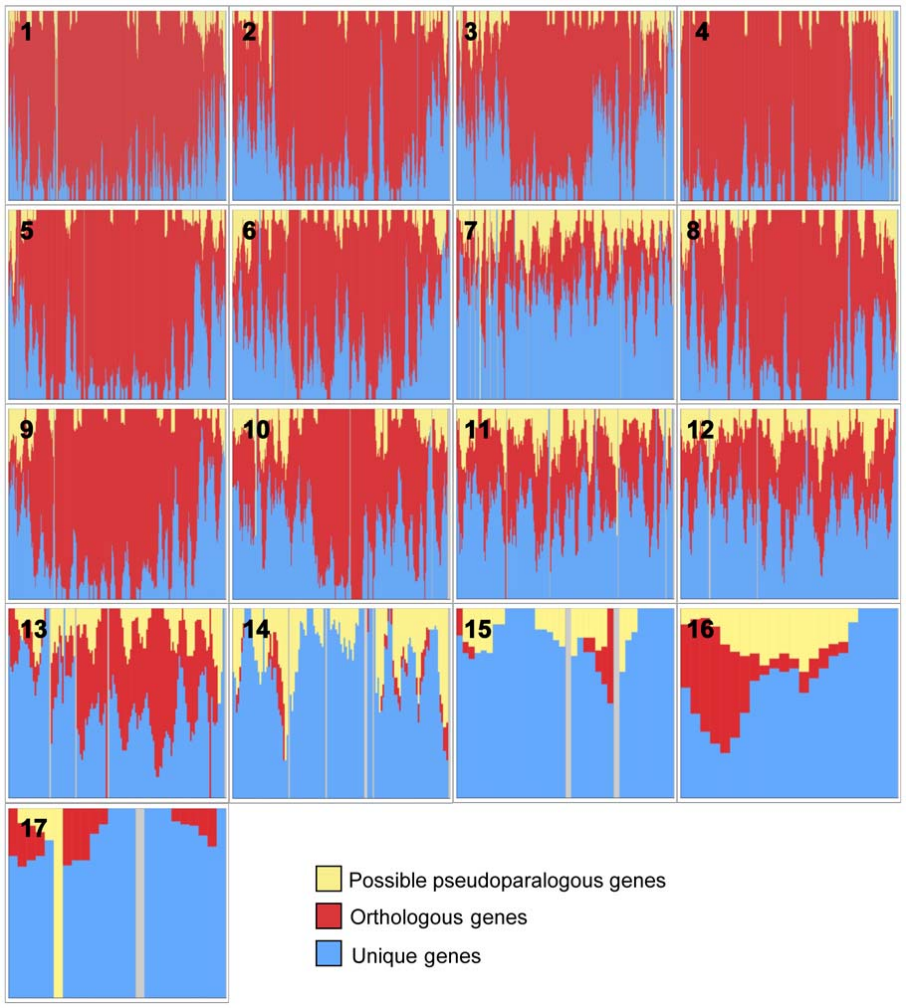
Distribution of orthologs, possible pseudoparalogs, and unique genes on each of the chromosomes of *N. haematococca* MPVI. Compositional statistical analysis using an additive log-ratio transformation [102] reveals that the distribution of genes within the three classes is statistically different on chromosomes 14, 15, and 17 than on the other chromosomes (p = 1.05e-5). Compositional statistical analysis using an additive log-ratio transformation [102] reveals that the distribution of genes within the three classes is statistically different on chromosomes 14, 15, and 17 than on the other chromosomes (p = 1.05e-5).

The genomic islands of *A. fumigatus* tend to be located in the sub-telomeric regions of chromosomes. In other fungi, genes involved in specific habitat associations, including pathogenicity, also are often located in the sub-telomeric regions [78]. In *N. haematococca* MPVI, gene placements differ among the chromosomes. Chromosomes 1, 4, 5, and 9 have most of their unique genes in the sub-telomeric regions (Figure 9). On the other large chromosomes, the unique genes are distributed in clusters throughout the chromosome. Chromosome 7 is unusual in that it is a large chromosome (3.0 Mb) that has some properties of the smaller chromosomes. For example, the majority of the genes on chromosome 7 are unique to *N. haematococca* MPVI (Figure 9). Chromosome 7 also has the largest number of possible pseudoparalogs (159) of all the chromosomes (Figure 4).

Only one isolate of *N. haematococca* MPVI (77-13-4) was sequenced in the current study and it is a third generation progeny from a laboratory cross between two field isolates. In *N. crassa*, where RIP was first defined, both RIP and the translocation of chromosomal fragments occur during the sexual cycle [79]. Thus, the crossing of the original isolates of *N. haematococca* MPVI might have increased the degree of RIP and/or affected the locations of genes in 77-13-4. An examination of field isolates of this fungus has revealed a high degree of chromosomal polymorphism and differences in the locations of studied genes [80]. Thus, it would be of great interest to determine the sequence of field isolates of *N. haematococca* MPVI and other species within the *F. solani* species complex and to compare the sizes and organization of their genomes to *F. graminearum* and to other fusaria in the *Gibberella* clade.

## Materials and Methods

### Isolates

*N. haematococca* MPVI isolates 77-13-4 (FGSC 9596, Fungal Genetics Stock Center) and 77-13-7 are ascospore isolates from a third generation cross between two field isolates: one (T2) obtained from a infected pea plant in NY and the other (T219) obtained from soil in a potato field in PA [81],[82]. Isolates HT1 and B-33 were derived from isolates 77-13-4 and 77-13-7, respectively, after treatment with benomyl and selection for the loss of chromosome 14 [35],[49].

### DNA isolation and sequencing

High molecular weight genomic DNA was isolated from protoplasts of 77-13-4 prepared by treatment of mycelia with a combination of lytic enzymes as described previously [83]. Whole-genome shotgun libraries (3-kb, 8-kb, and 40-kb DNA inserts) of 77-13-4 were constructed and sequenced as previously described [84].

### RNA isolation and sequencing

Two cDNA libraries were constructed and sequenced to facilitate the automated gene calling programs. The RNA for the cDNA libraries was obtained from mycelia treated in two different ways: both were grown in a rich medium, potato dextrose broth, (PDB) (Difco, Sparks, MD) and one of these was treated with pisatin. Spores of 77-13-4 (10^5^ ml^−1^) from V8 agar medium [85] were added to 100 ml of PDB in a 250 ml Erlenmeyer flask and grown at room temperature on a rotary shaker (180 rpm) for 24 h. The mycelium was collected by vacuum filtration and stored at −80°C. Mycelium for the pisatin-treated library was collected after overnight growth in PDB, washed in 10 ml of 0.7 M NaCl, and added to 50 ml of phosphate buffer (50 mM potassium phosphate buffer, pH 6.5). After 2 hours, pisatin in DMSO was added to a final concentration of 30 µg of pisatin/ml and 1% DMSO and after another 4.5 hours the mycelium was collected and frozen at −80°C.

For RNA isolation the mycelia were lyophilized and ground under liquid nitrogen using a mortar and pestle. RNA was isolated as described in Mandel et al. [86] with the exception that ground mycelia (200 mg) were placed in an RNase-free 2-ml tube, to which 0.7 ml of ice-cold breaking buffer was added, the mycelia were suspended, and 0.6 ml of acid phenol added. cDNA libraries were constructed and sequenced as described previously with minor differences that include: the size ranges of cDNA (0.6 k–2 kb and >2 kb), the cloning vector (pMCL200cDNA), and the sequencing primers (Fw: 5′-AGGAAACAGCTATGACCA-3′, Rv: 5′-GTTTTCCCAGTCACGACGTTGTA-3′) [87]. 24,793 ESTs were obtained from mycelium grown in the PDB medium and 8,327 from the mycelium treated with pisatin.

### Genome finishing methods

Initial read layouts from the whole genome shotgun assembly were converted into a Phred/Phrap/Consed pipeline [88] and, following manual inspection of the assembled sequences, finishing was performed by resequencing plasmid subclones and by walking on plasmid subclones or fosmids using custom primers. All finishing reactions were performed with 4∶1 BigDye to dGTP BigDye terminator chemistry (Applied Biosystems). Repeats in the sequence were resolved by transposon-hopping 8-kb plasmid clones. Fosmid clones were shotgun sequenced and finished to fill large gaps, resolve large repeats, and to extend into chromosome telomere regions where possible. After finishing, the genome remained in 209 scaffolds as a result of many regions of the genome being apparently unclonable in the shotgun libraries constructed for this project.

The resulting assembly was joined and validated by alignments to a *N. haematococca* optical map (generated by digestion with *Nhe*I), with 92.26% of the sequence (72 scaffolds or 47,191,137 bp) being placed onto 17 chromosome optical maps. 3,958,438 bp of the 137 smaller scaffolds remain unplaced because of the lack of sufficient restriction sites. The genome consists of 51,149,575 base pairs of finished sequence with an estimated error rate of less than 1 error in 100,000 bp.

### Optical mapping

Protoplasts of 77-13-4, made as described above [83], were directly lysed with a protoplast lysing solution (10 mM EDTA, 5 mM EGTA, 1 mg/ml proteinase K, pH 8.0) by heating to 65°C for 30 min to 1 hr and then incubating overnight at 37°C. The protoplast concentration was adjusted to ∼700 protoplasts per microliter. Optical mapping operations followed previously published techniques [89]; briefly, randomly sheared high molecular weight DNA was loaded onto optical mapping chips for restriction digestion by *Nhe*I (New England Biolabs). DNA was stained with YOYO-1 fluorochrome (Invitrogen) and the chips were scanned on an automated fluorescence microscope system for image capture, analysis, and map construction [90]. Resulting single-molecule restriction maps were assembled into genome-wide contigs [91],[92] that served as map scaffolds for sequence joining and validation efforts.

### Gene prediction and automated annotation

Gene models (15,707) were predicted and automatically annotated using the Joint Genome Institute (JGI) Annotation Pipeline. Several gene predictors were used on repeat masked assembly: ab initio FGENESH and homology-based FGENESH+ [93] and Genewise [94]. The predicted gene models were verified, corrected, and extended using 33,142 *N. haematococca* MPVI ESTs. All predicted gene models were functionally annotated by homology to proteins from the NCBI non-redundant set and classified according to Gene Ontology [95], eukaryotic orthologous groups (KOGs [96]), and KEGG metabolic pathways [97]. Of the 15,707 models, 93% were complete models, 25% were supported with EST aligment, 94% with NR alignment, 73% with Swissprot alignment and 52% with Pfam alignments. For every locus the ‘best’representative model was selected based on EST and homology support, to produce a non-redundant representative set, subject to manual curation and the analysis described here.

### Best fit analysis of *N. haematococca* to other fungi

Each predicted protein from the *N. haematococca* MPVI genome was used as a query in a TBLASTN search of a database consisting of the genomes of *A. oryzae*, *A. nidulans*, *C. globosum*, *C. immitis*, *F. graminearum*, *M. oryzae*, *N. crassa*, and *S. cerevisiae*. For each protein query, the genome with the best hit below a threshold of 1e-5 was identified.

### Phylogenetic analysis

The predicted amino acid sequences for hypothetical proteins were aligned with ClustalW 1.81 [98]. The resulting alignment files were imported into MacClade 4.08 [99] for manual editing and exclusion of all ambiguously aligned regions. Heuristic searches for maximum parsimony (MP) were conducted in PAUP* [100], and neighbor-joining distance trees were generated in MacVector 10.0.2 (Symantec Corporation). Statistical support was calculated from 1,000 bootstrap replicates.

### RIP index

To identify repetitive regions of the genome in the absence of a curated repeat library, the genome was divided into 1 kb non-overlapping windows and BLASTN was used to align each against the complete genome. If the only match greater than 500 bp was a self-match, then the window was labeled as unique, otherwise it was labeled as repetitive. For each 1 kb window, the ratio of TpA/ApT frequency was calculated.

### Experimental demonstration of RIP

A transformant (Tr78.2) of *N. haematococca* that contained several copies of the *hph* gene from the transformation vector pCWHyg1 [101], was crossed (cross 370) with 77-15-7 as previously described [82]. The *hph* gene is linked to the homoserine utilization phenotype (HUT) in Tr78.2 and 370 progeny were screened for hygromycin sensitivity and HUT. All forty progeny from cross 370 were sensitive to hygromycin, and half were HUT^+^. DNA was isolated from two hygromycin sensitive/HUT^+^ isolates (370-4 and 370-8) as previously described [32]. Sequences of *hph* were amplified using PCR and the primers Hyg-F (5′-CGGAGATTCGTCGTTCTGAAGAG-3′) and Hyg-R (5′-TTCTACACAGCCATCGGTCCAG-3′) following the manufacturer's protocol (Invitrogen) and the following set of conditions (94°C for 45 sec, 59°C for 30 sec, 72°C for 1.5 min, for 35 cycles). The resulting 1,242 bp products containing *hph* were cloned into the pGEM T-EZ vector (Promega Corporation, Madison, WI) and *hph* was sequenced using the Hyg2-F (5′-ACGCGACAACTGAGTGACTG-3′) primer adjacent to *hph*. Sequencing was done by the Genomic Analysis and Technology Core (GATC) facility at the University of Arizona.

### Pulsed-field gel electrophoresis (PFGE) analyses of chromosome-sized DNA

77-13-7 and its derivative B-33 have been described [49], as have 77-13-4 and its derivative HT-1 [35]. The preparation of chromosome-sized DNAs and the PFGE were performed essentially as described previously [35],[72]. For making protoplasts of 77-13-7 and B-33, the enzyme mixture of Garmaroodi and Taga [72] was used, while the enzyme mixture of Rodriguez-Carres et al. [35] was used for 77-13-4 and HT-1. Protoplasts (ca. 3×10^8^ protoplasts/ml) were embedded in low melting temperature agarose (Bio-Rad Laboratories Inc., Hercules, CA) and chromosome-sized DNAs were separated in 0.5× TBE buffer on a 1% (w/v) pulsed field certified agarose (Bio-Rad Laboratories) gel with a contour-clamped homogeneous electric field apparatus (CHEF-DR II, Bio-Rad Laboratories). Running conditions were 5.4 V/cm and pulse time of 120 s for 13 h followed by 180 s for 13 h. Chromosomal DNAs of *S. cerevisiae* (Bio-Rad Laboratories) were used as the size markers.

### Attempts to detect chromosome-specific sequences found on the ends of supernumerary chromosomes

Genomic DNA of *N. haematococca* MPVI was isolated as previously described [83]. PCR was used to test for the presence of sequences found on the ends of the assembled sequences representing chromosomes 14, 15, and 17 in isolates 77-13-7, 77-13-4, B-33, and HT-1. Primers were designed to amplify regions of scaffolds located on the ends of the respective chromosomes: scaffolds 24 and 62 for chromosome 14, scaffolds 25 and 57 for chromosome 15, and scaffolds 74 and 90 for chromosome 17. These sequences were blasted against the *N. haematococca* MPVI genome sequence to confirm that they were not found on chromosomes other than those of interest.

Sequences of the primers (Invitrogen) used for PCR were as follows: for chromosome 14, scaffold 24: forward primer, 5′-GCCAGGAGATCGAGATATGA-3′ and reverse primer, 5′-GTGGATGAGATCGGTGTTTC-3′; for chromosome 14, scaffold 62: forward primer, 5′-CTCCATCTTCTCGGCAATGT-3′ and reverse primer, 5′-CTTGGTTCACTCGCATACTTG-3′; for chromosome 15, scaffold 25: forward primer, 5′-GACCGTCAAGGGAGCTACAG-3′ and reverse primer, 5′-ATCAGGGGTCATGTGAAGC-3′; for chromosome 15, scaffold 57: forward primer, 5′-GGCCTTTGTACTCGCATTTA-3′ and reverse primer, 5′-GACCCTCTGCCTTCTTCTTC-3′; for chromosome 17, scaffold 74: forward primer, 5′- CGCCCACTTCTTTGTCTCTA-3′ and reverse primer, 5′-AGCGAATTCATTTGAAGCAG -3′; and for chromosome 17, scaffold 90: forward primer, 5′-GGAGACGTTGATGAGATTGG -3′ and reverse primer, 5′-CATCTGTTGAACCCACACAA -3′. Each reaction had a total volume of 50 µl containing 300 nM forward primer, 300 nM reverse primer, 1 µl (∼50 ng) DNA template, 5 mM MgCl_2_, 5 µl 10× PCR buffer, 200 µM (each) of dATP, dTTP, dCTP, and dGTP, and 1 unit Taq DNA Polymerase. The PCR protocol consisted of an initial denaturation step of 95°C for 3 min., 35 cycles at 95°C for 30 sec, 55°C for 30 sec, and 72°C for 30 sec, and a final elongation step at 72°C for 30 sec. PCR products were run on a 0.8% agarose gel containing ethidium bromide and visualized under UV light. Genomic DNA of *N. haematococca* isolates 77-13-4 and 77-13-7 was used as positive controls.

### Search for segmental duplications

BlastP searches of the protein set against itself with a threshold value of 1e-20 identified 2,259 gene pairs as best-bidirectional hits. Segmental duplicated regions were defined as genomic regions that share at least three genes in the same order and orientation, while the distance between neighboring gene pairs is less than 50 kb.

## Supporting Information

Figure S1 Ratio of the number of genes in gene families in *N. haematococca* MPVI versus *F. graminearum*. Only gene families that had ≥10 members in *N. haematococca* MPVI were used in the analysis. The number of genes per family was derived from Interpro calls made by the JGI for *N. haematococca* MPVI, and by the Munich Institute for Protein Sequences (MIPS) for *F. graminearum*.
(1.27 MB TIF)

Figure S2 The CAX (calcium exchanger) transporter clade from select fungal genomes. Maximum parsimony analysis was used to establish the phylogenetic relationship between the ortholog (Nh65123, red box) and the pseudoparalog (Nh101770, blue box) of *N. haematococca* MPVI.
(8.06 MB TIF)

Figure S3 Distibution of repeat identity. NC7 is *N. crassa*, MG5 is *M. oryzae*, AN1 is *A. nidulans*, “FG3 Repeats” is *F. graminearum* and “FS Repeats” is *N. haematococca* MPVI.
(4.09 MB TIF)

Figure S4 Effect of RIP on a family of telomere-associated helicases (TAH) in *N. haematococca* MPVI. Partial alignment of the 12 predicted TAH genes (*tah*), spanning only the first three conserved motifs. The top row shows the predicted translation of the fourth *tah* gene on scaffold 45 (TAH_45-4). While many mutations occur in the wobble position, note the presence of nonsense codons (*). Nucleotides in red (G to A change) and orange (C to T change) can be explained by a single RIP-type mutation, while nucleotides in pink denoted non-RIP-type transversions. Conversion of RIP-type C∶G to T∶A mutations back to the likely original sequence (“de-RIP”, blue), results in a consensus sequence (TAH_ORI) that closely resembles that of the *Metarhizium anisopliae TAH1* sequence (MaTAH1; note absence of nonsense codons in the derived consensus sequence, residues in red indicate changes compared to the TAH_45-4 sequence). De-RIP of the complete coding region results in a single large ORF without predicted introns or nonsense codons, similar to the *M. anisopliae TAH1* gene (Inglis PW, Rigden DJ, Mello LV, Louis EJ, Valadares-Inglis MC 2005 Monomorphic subtelomeric DNA in the filamentous fungus, Metarhizium anisopliae, contains a RecQ helicase-like gene. Mol Genet Genomics 274: 79–90).
(7.54 MB TIF)

Figure S5 Repeat-induced point mutation (RIP) in *N. haematococca* MPVI. The hygromycin resistance (*hph*) gene is mutated from G to A at multiple TpG positions (indicated in red) in isolates 370-4 and 370-8.
(1.36 MB TIF)

Table S1 Comparison of genome statistics of several filamentous ascomycete fungi.
(0.06 MB DOC)

Table S2 Properties of the genes of *N. haematococca* MPVI.
(0.03 MB DOC)

Table S3 Gene families that are at least two-fold larger in *Nectria haematococca* MPVI than in *Fusarium graminearum*.
(0.09 MB DOC)

Table S4 The number of ABC transporters in *Nectria haematococca* MPVI compared to other fungi.
(0.05 MB DOC)

Table S5 Carbohydrate-active enzymes in *N. haematococca* MPVI compared to other fungi.
(0.07 MB DOC)

Table S6 The number of cytochrome P450 genes in *Nectria haematococca* MPVI compared to other fungi.
(0.06 MB DOC)

Table S7 Number of predicted genes in *Nectria haematococca* MPVI that contain transcription factor motifs compared to other fungi.
(0.10 MB DOC)

Table S8 The number of chromatin genes in *N. haematococca* MPVI compared to other fungi.
(0.05 MB DOC)

Table S9 Distribution of repeat elements in the genome of *Nectria haematococca* MPVI.
(0.08 MB DOC)

Table S10 Properties of the chromosomes and genes on each chromosome in *N. haematococca* MPVI.
(0.09 MB DOC)

Table S11 The protein kinases of *N. haematococca* MPVI compared to *S. cerevisiae*.
(0.03 MB DOC)

Table S12 The number of polyketide synthases (PKS) and nonribosomal peptide synthetases (NRPS) of Nectria haematococca MPVI compared to other fungi.
(0.06 MB DOC)

Table S13 Distribution of Small Secreted Proteins (SSP) among filamentous Ascomycetes as identified by SignalP.
(0.05 MB DOC)
