# Supplementary figures and images for: The Genome of Nectria haematococca: Contribution of Supernumerary Chromosomes to Gene Expansion
Source: PLoS Genet. 2009 Aug 28;5(8):e1000618. doi: 10.1371/journal.pgen.1000618 (PMC2725324; doi:10.1371/journal.pgen.1000618)

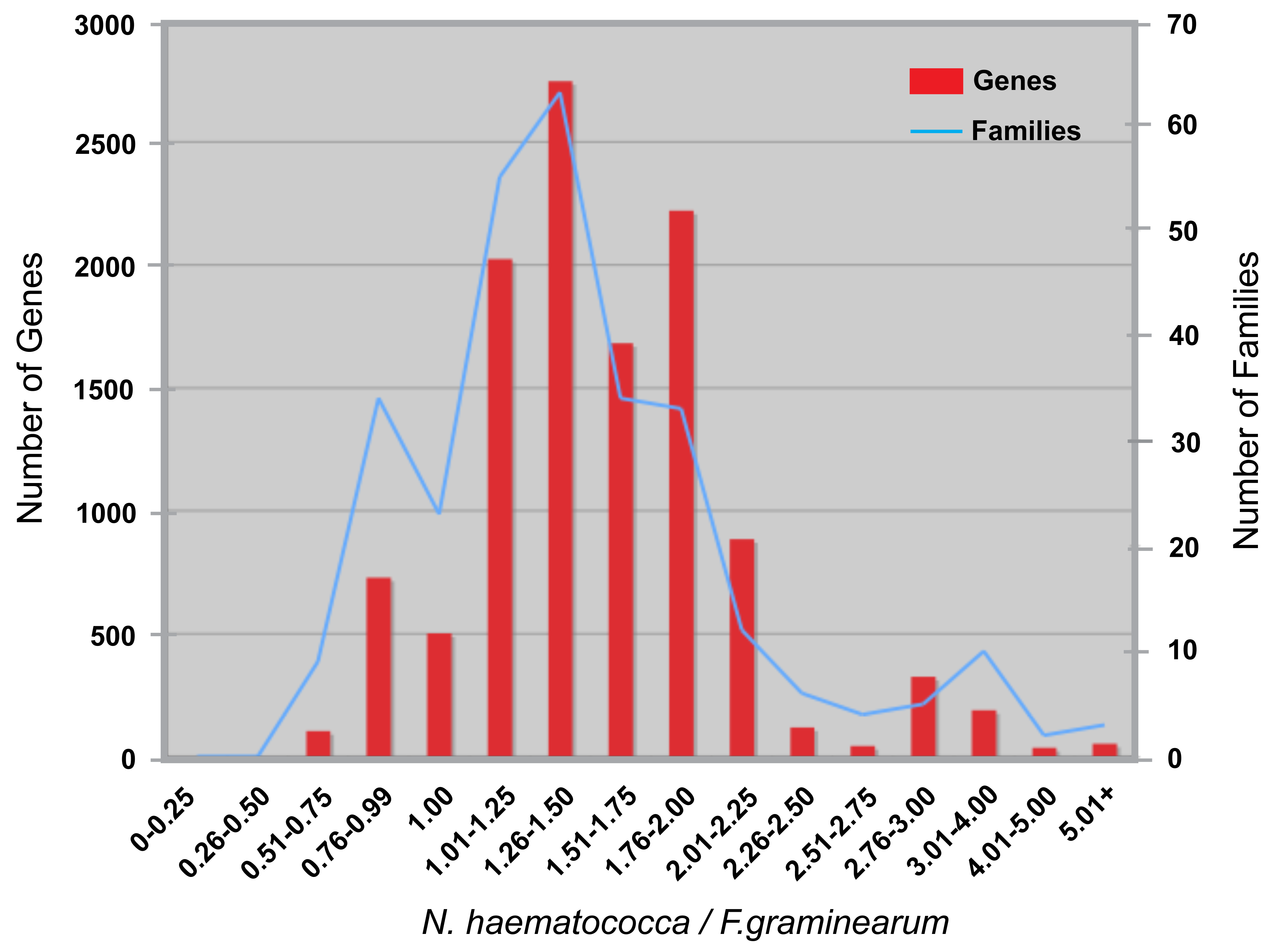

Supplement: Figure S1 — Ratio of the number of genes in gene families in N. haematococca MPVI versus F. graminearum. Only gene families that had ≥10 members in N. haematococca MPVI were used in the analysis. The number of genes per family was derived from Interpro calls made by the JGI for N. haematococca MPVI, and by the Munich Institute for Protein Sequences (MIPS) for F. graminearum. (1.27 MB TIF) [file pgen.1000618.s001.tif]

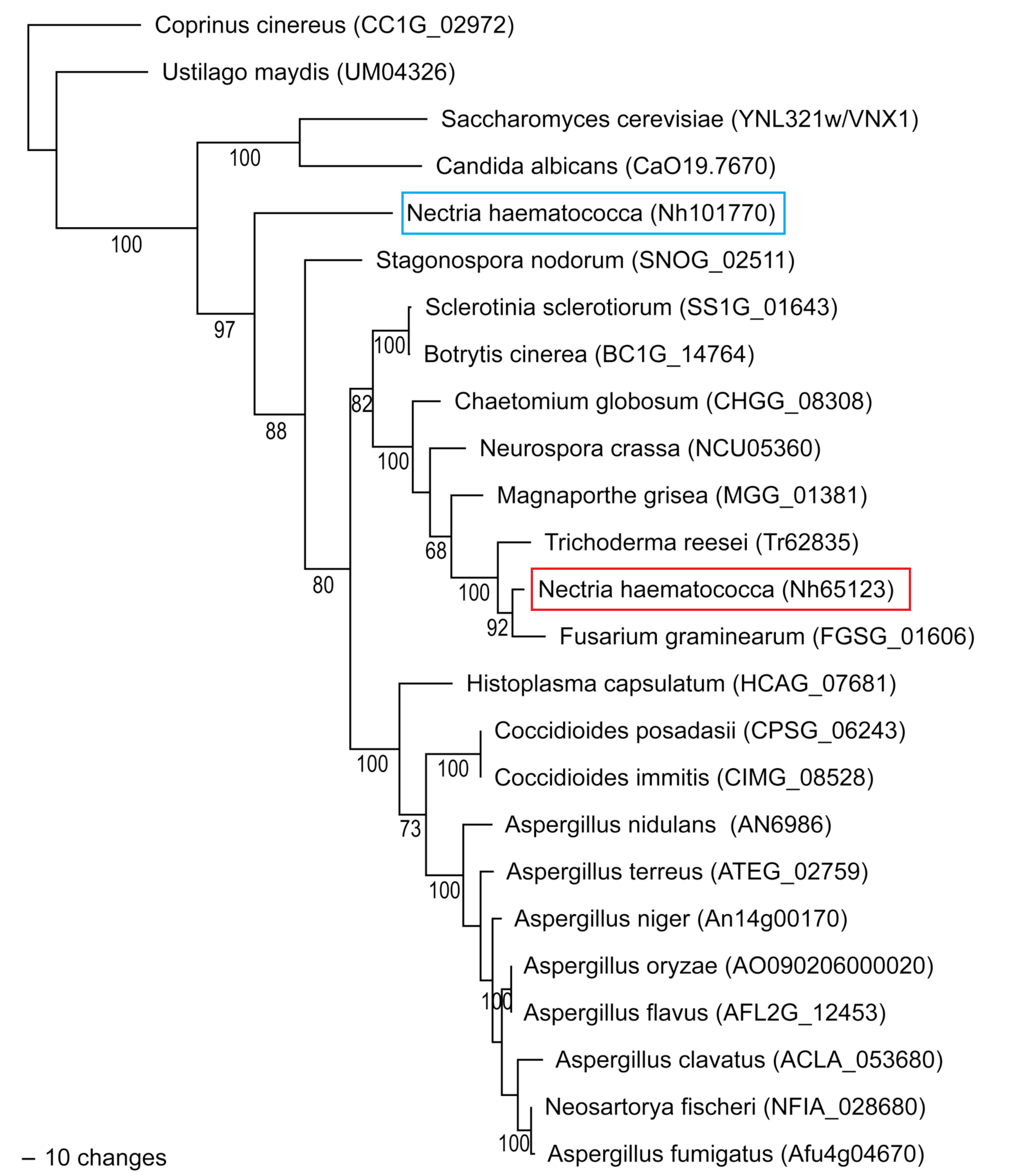

Supplement: Figure S2 — The CAX (calcium exchanger) transporter clade from select fungal genomes. Maximum parsimony analysis was used to establish the phylogenetic relationship between the ortholog (Nh65123, red box) and the pseudoparalog (Nh101770, blue box) of N. haematococca MPVI. (8.06 MB TIF) [file pgen.1000618.s002.tif]

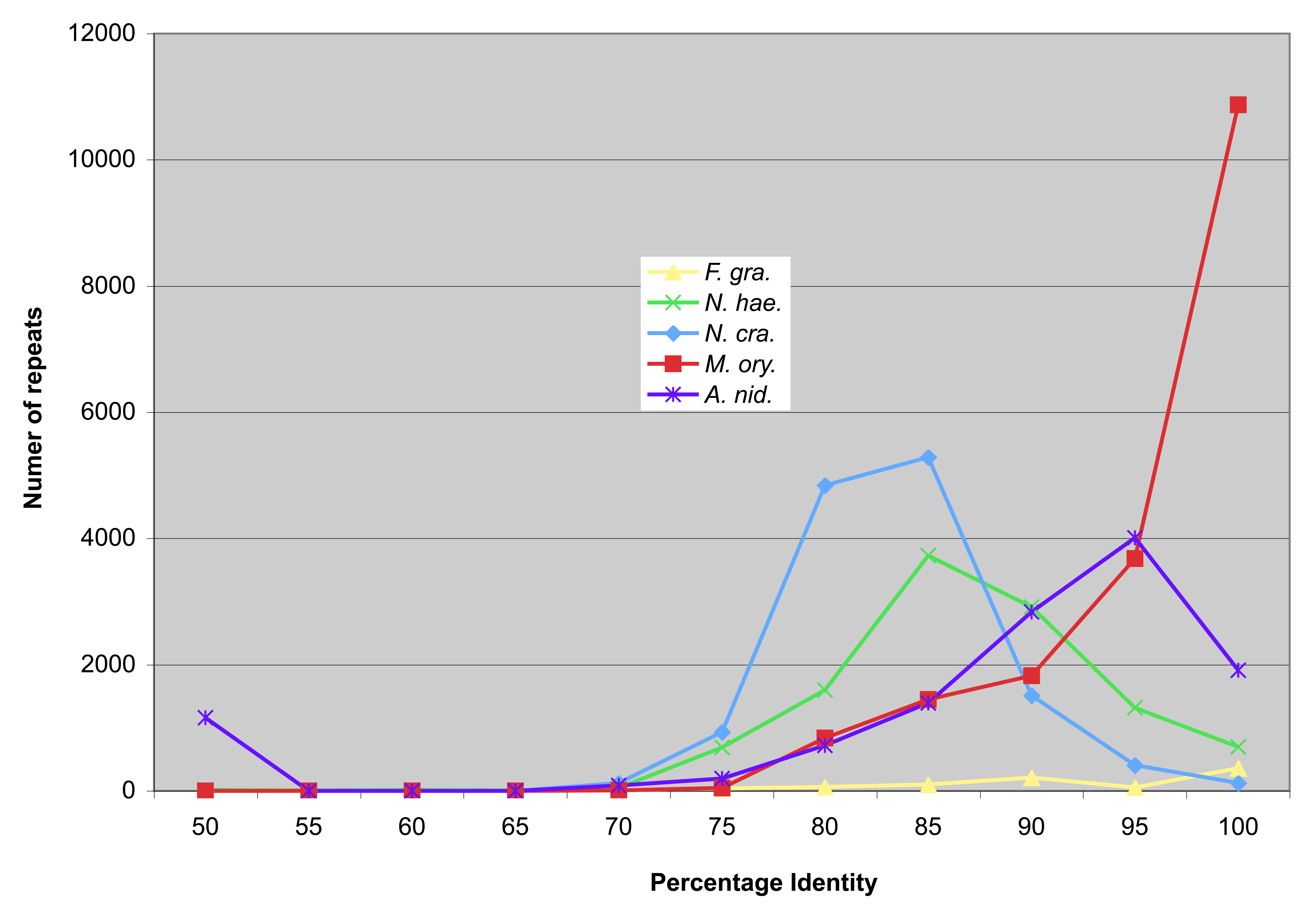

Supplement: Figure S3 — Distibution of repeat identity. NC7 is N. crassa, MG5 is M. oryzae, AN1 is A. nidulans, “FG3 Repeats” is F. graminearum and “FS Repeats” is N. haematococca MPVI. (4.09 MB TIF) [file pgen.1000618.s003.tif]

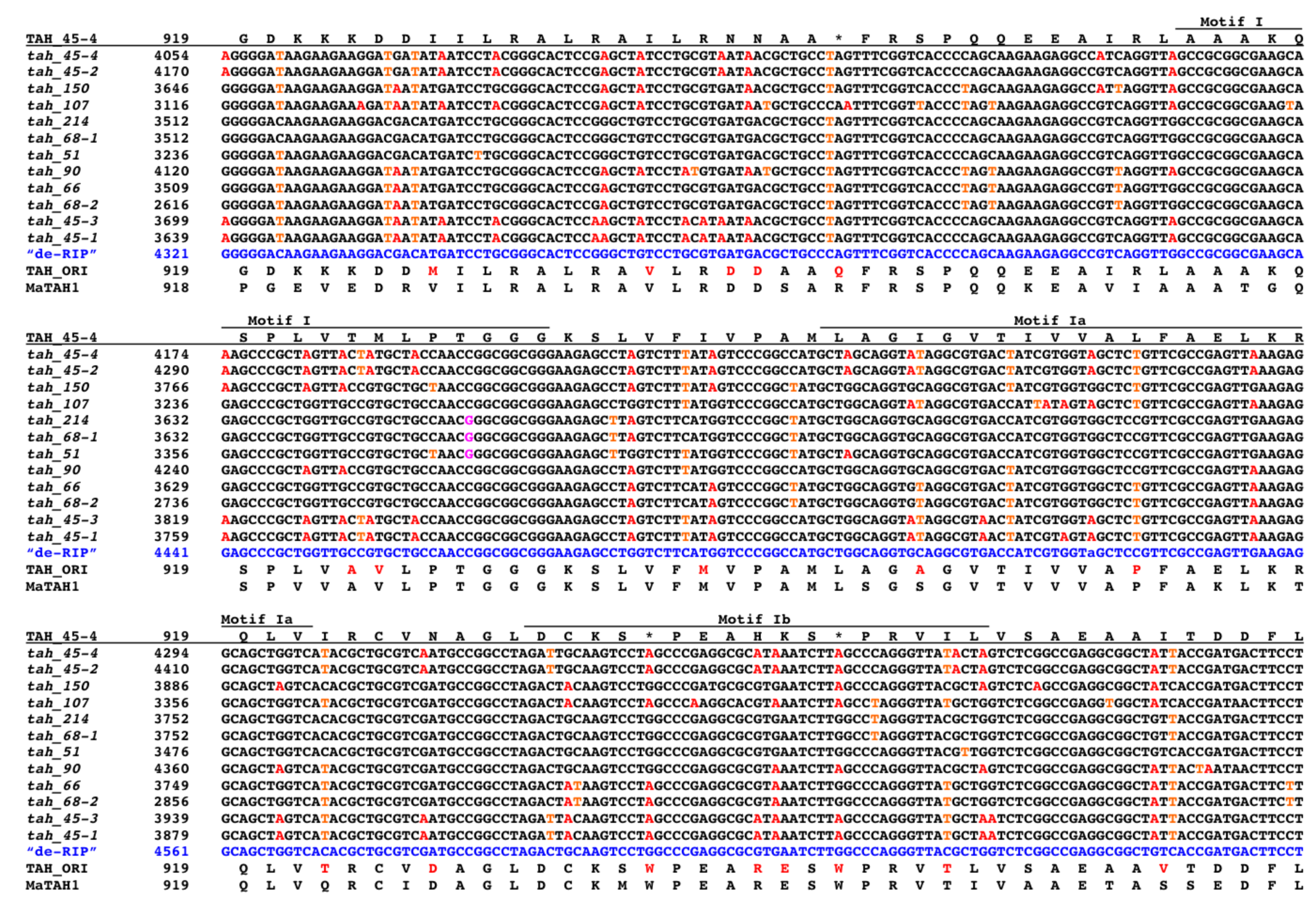

Supplement: Figure S4 — Effect of RIP on a family of telomere-associated helicases (TAH) in N. haematococca MPVI. Partial alignment of the 12 predicted TAH genes (tah), spanning only the first three conserved motifs. The top row shows the predicted translation of the fourth tah gene on scaffold 45 (TAH_45-4). While many mutations occur in the wobble position, note the presence of nonsense codons (*). Nucleotides in red (G to A change) and orange (C to T change) can be explained by a single RIP-type mutation, while nucleotides in pink denoted non-RIP-type transversions. Conversion of RIP-type C∶G to T∶A mutations back to the likely original sequence (“de-RIP”, blue), results in a consensus sequence (TAH_ORI) that closely resembles that of the Metarhizium anisopliae TAH1 sequence (MaTAH1; note absence of nonsense codons in the derived consensus sequence, residues in red indicate changes compared to the TAH_45-4 sequence). De-RIP of the complete coding region results in a single large ORF without predicted introns or nonsense codons, similar to the M. anisopliae TAH1 gene (Inglis PW, Rigden DJ, Mello LV, Louis EJ, Valadares-Inglis MC 2005 Monomorphic subtelomeric DNA in the filamentous fungus, Metarhizium anisopliae, contains a RecQ helicase-like gene. Mol Genet Genomics 274: 79–90). (7.54 MB TIF) [file pgen.1000618.s004.tif]

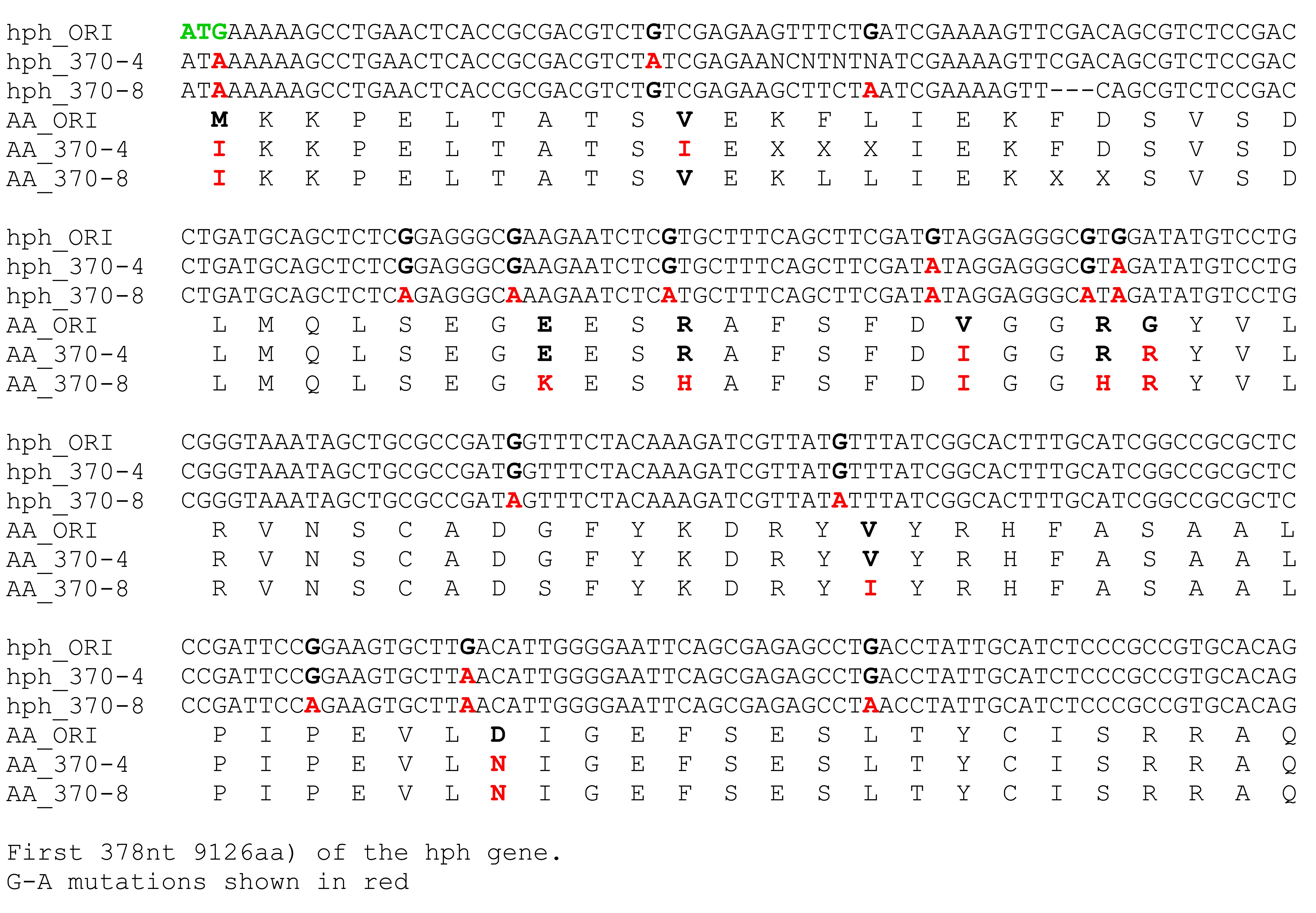

Supplement: Figure S5 — Repeat-induced point mutation (RIP) in N. haematococca MPVI. The hygromycin resistance (hph) gene is mutated from G to A at multiple TpG positions (indicated in red) in isolates 370-4 and 370-8. (1.36 MB TIF) [file pgen.1000618.s005.tif]
