# Supplementary material for: The Genome of Nectria haematococca: Contribution of Supernumerary Chromosomes to Gene Expansion
Source: PLoS Genet. 2009 Aug 28;5(8):e1000618. doi: 10.1371/journal.pgen.1000618 (PMC2725324; doi:10.1371/journal.pgen.1000618)
Supplement: Table S1 — Comparison of genome statistics of several filamentous ascomycete fungi. (0.06 MB DOC) [file pgen.1000618.s006.doc]

**Table S1.** Comparison of genome statistics of several filamentous ascomycete fungi.

| Fungal Species | Strain | Genome Size (Mb) | Number of ORFs |
| --- | --- | --- | --- |
|  |  |  |  |
| *Alternaria brassicicola*^a^ | ATCC 96866 | 30.3 | 10,688 |
| *Aspergillus clavatus*^b^ | NRRL 1 | 27.86 | 9,121 |
| *Aspergillus flavus*^b^ | NRRL 3357 | 36.79 | 12,604 |
| *Aspergillus fumigatus*^b^ | Af293 | 29.38 | 9,887 |
| *Aspergillus nidulans*^b^ | FGSC A4 | 30.07 | 10,701 |
| *Aspergillus niger*^b^ | ATCC 1015 | 37.2 | 11,200 |
| *Aspergillus oryzae*^b^ | ATCC 42149 | 37.12 | 12,336 |
| *Aspergillus terreus*^b^ | NIH 2624 | 29.33 | 10,406 |
| *Chaetomium globosum*^b^ | CBS 148.51 | 34.89 | 11,124 |
| *Coccidioides immitis*^b^ | RS | 28.89 | 10,355 |
| *Coccidioides posadasii*^b^ | Silveira | 27.47 | 10,061 |
| *Cryphonectria parasitica*^a^ | EP 155 | 43.9 | 11,184 |
| *Fusarium graminearum*^b^ | PH-1 | 36.45 | 13,332 |
| *Magnaporthe oryzae*^b^ | 70-15 | 41.7 | 11,074 |
| ***Nectria haematococca* MPVI** | **77-13-4** | **51.15*** | **15,707** |
| *Neosartorya fischeri*^b^ | NRRL 181 | 32.55 | 10,406 |
| *Neurospora crassa*^b^ | OR74A | 39.23 | 9,826 |
| *Sclerotinia sclerotiorum*^b^ | 1980 | 38.33 | 14,522 |
| *Stagonospora nodorum*^b^ | SN15 | 37.24 | 16,597 |
| *Trichoderma reesei*^a^ | QM 6a | 34.1 | 9,129 |
| *Verticilli um dahliae*^b^ | VdLs.17 | 33.83 | 10,535 |
|  |  |  |  |

***** Based on the assembled genome sequence. Based on optical map data, the size of *N. haematococca* MPVI genome is 54.43 Mb.

^a,b^ All fungal genome sequence data was publically available from the ^a^Joint Genome Institute (http://genome.jgi-psf.org/) or the ^b^Broad Fungal Genome Initiative (http://www.broad.mit.edu/node/304).
