# Supplementary material for: The Genome of Nectria haematococca: Contribution of Supernumerary Chromosomes to Gene Expansion
Source: PLoS Genet. 2009 Aug 28;5(8):e1000618. doi: 10.1371/journal.pgen.1000618 (PMC2725324; doi:10.1371/journal.pgen.1000618)
Supplement: Table S2 — Properties of the genes of N. haematococca MPVI. (0.03 MB DOC) [file pgen.1000618.s007.doc]

**Table S2.** Properties of the genes of *N. haematococca* MPVI

| **Property** | **Estimated value*** |
| --- | --- |
|  |  |
| Number of genes | 15,707 |
| Gene density (genes/Mb) | 307 |
| Gene length (bases) | 1,674 |
| Transcript length (bases) | 1,503 |
| Protein length (amino acids) aacidsacids) | 480 |
| Number of exons/gene | 3.1 |
| Exon length (bases) | 488 |
| Intron length (bases) | 84 |
|  |  |

*The calculations are based in version 2 (v2) of the assembly of 6/2006 of the genome sequence of *N. haematococca* MPVI. Except for the number of genes, the values are the averages for each property.
