# Supplementary material for: The Genome of Nectria haematococca: Contribution of Supernumerary Chromosomes to Gene Expansion
Source: PLoS Genet. 2009 Aug 28;5(8):e1000618. doi: 10.1371/journal.pgen.1000618 (PMC2725324; doi:10.1371/journal.pgen.1000618)
Supplement: Table S3 — Gene families that are at least two-fold larger in Nectria haematococca MPVI than in Fusarium graminearum. (0.09 MB DOC) [file pgen.1000618.s008.doc]

**Table S3.** Gene families that are at least two-fold larger in *Nectria haematococca* MPVI than in

*Fusarium graminearum.*

| **Interpro Family Name** | **Gene Number*** | |  | **Ratio** |
| --- | --- | --- | --- | --- |
|  | ***N. hae.*** | ***F. gra.*** |  | ***N.hae.*/*F. gra. ggragra.*** |
|  |  |  |  |  |
| Isoflavone reductase | 30 | 2 |  | 15.00 |
| Blue (type 1) copper domain | 10 | 1 |  | 10.00 |
| Retinal pigment epithelial membrane protein | 13 | 2 |  | 6.50 |
| Bacterial ring hydroxylating dioxygenase, alpha subunit | 10 | 2 |  | 5.00 |
| Glycoside hydrolase, family 43 | 26 | 6 |  | 4.33 |
| YjgF-like protein | 16 | 4 |  | 4.00 |
| Class II aldolase/adducin, N-terminal | 12 | 3 |  | 4.00 |
| FAD dependent oxidoreductase | 19 | 5 |  | 3.80 |
| 1-aminocyclopropane-1-carboxylate synthase | 11 | 3 |  | 3.67 |
| Monooxygenase, FAD-binding | 21 | 6 |  | 3.50 |
| Peptidase S15 | 14 | 4 |  | 3.50 |
| Tyrosine protein kinase, active site | 36 | 11 |  | 3.27 |
| L-carnitine dehydratase/bile acid-inducible protein F | 13 | 4 |  | 3.25 |
| Tetracycline resistance protein | 16 | 5 |  | 3.20 |
| Endoribonuclease L-PSP | 31 | 10 |  | 3.10 |
| Protein of unknown function DUF636 | 18 | 6 |  | 3.00 |
| Cys/Met metabolism pyridoxal-phosphate-dependent enzymes | 12 | 4 |  | 3.00 |
| Phytanoyl-CoA dioxygenase | 12 | 4 |  | 3.00 |
| N/apple PAN | 17 | 6 |  | 2.83 |
| Heterokaryon incompatibility | 266 | 94 |  | 2.83 |
| Mandelate racemase/muconate lactonizing enzyme | 11 | 4 |  | 2.75 |
| Hydantoinase/oxoprolinase | 11 | 4 |  | 2.75 |
| Ketopantoate reductase ApbA/PanE | 11 | 4 |  | 2.75 |
| snRNP domain | 11 | 4 |  | 2.75 |
| Flavin-containing amine oxidase | 10 | 4 |  | 2.50 |
| Isopenicillin N synthase | 10 | 4 |  | 2.50 |
| Hydantoinaseoxoprolinase, N-terminal | 10 | 4 |  | 2.50 |
| Beta-lactamase-like | 31 | 13 |  | 2.38 |
| Beta-lactamase | 35 | 15 |  | 2.33 |
| FMN-dependent alpha-hydroxy acid dehydrogenase | 23 | 10 |  | 2.30 |
| Alpha/beta hydrolase | 27 | 12 |  | 2.25 |
| Glycoside hydrolase, family 5 | 22 | 10 |  | 2.20 |
| Glycosyl transferase, family 28 | 11 | 5 |  | 2.20 |
| HpcH/HpaI aldolase | 11 | 5 |  | 2.20 |
| Glycoside hydrolase, family 2, sugar binding | 11 | 5 |  | 2.20 |
| Flavoprotein monooxygenase | 91 | 42 |  | 2.17 |
| Aromatic-ring hydroxylase | 97 | 45 |  | 2.16 |
| Oxidoreductase, N-terminal | 32 | 15 |  | 2.13 |
| Peptidase S10, serine carboxypeptidase | 19 | 9 |  | 2.11 |
| Fungal specific transcription factor | 338 | 163 |  | 2.07 |
| Aflatoxin biosynthesis regulatory protein | 41 | 20 |  | 2.05 |
| Zinc-containing alcohol dehydrogenase superfamily | 155 | 76 |  | 2.04 |
| Sulfatase | 20 | 10 |  | 2.00 |
| Amine oxidase | 18 | 9 |  | 2.00 |
| Rieske [2Fe-2S] domain | 14 | 7 |  | 2.00 |
| Pyruvate decarboxylase | 12 | 6 |  | 2.00 |
| Intradiol ring-cleavage dioxygenase | 12 | 6 |  | 2.00 |
| Cyclin, N-terminal domain | 12 | 6 |  | 2.00 |
| GPR1/FUN34/yaaH | 10 | 5 |  | 2.00 |
| Isocitrate lyase and phosphorylmutase | 10 | 5 |  | 2.00 |
| Glyoxalase/Bleomycin resistance protein/dioxygenase domain | 10 | 5 |  | 2.00 |
| HAD-superfamily hydrolase, subfamily IA, variant 3 | 10 | 5 |  | 2.00 |
|  |  |  |  |  |

*Only gene families with ≥ 10 members were analyzed. *N. hae*. = *Nectria haematococca* MPVI;

*F. gra*. = *Fusarium graminearum*. The number of genes per family is derived from Interpro calls

made by the JGI for *N. haematococca* MPVI and by the Munich Institute for Protein Sequences

(MIPS) for *F. graminearum* (http://mips.gsf.de/projects/fungi/Fgraminearum.html).
