# Supplementary material for: The Genome of Nectria haematococca: Contribution of Supernumerary Chromosomes to Gene Expansion
Source: PLoS Genet. 2009 Aug 28;5(8):e1000618. doi: 10.1371/journal.pgen.1000618 (PMC2725324; doi:10.1371/journal.pgen.1000618)
Supplement: Table S4 — The number of ABC transporters in Nectria haematococca MPVI compared to other fungi. (0.05 MB DOC) [file pgen.1000618.s009.doc]

**Table S4.** The number of ABC transporters in *Nectria haematococca* MPVI compared to other fungi.

| **Fungal species** | **Number of ABC Transporters* Transporters** |
| --- | --- |
|  |  |
| *Aspergillus nidulans* | 45 |
| *Aspergillus fumigatus* | 45 |
| *Aspergillus oryzae* | 72 |
| *Coccidioides posadasii* | 30 |
| *Cryptococcus neoformans* | 29 |
| *Fusarium graminearum* | 54 |
| *Magnaporthe oryzae* | 50 |
| ***Nectria haematococca* MPVI** | **68** |
| *Neurospora crassa* | 31 |
| *Saccharomyces cerevisiae* | 24 |
| *Schizosaccharomyces pombe* | 9 |
|  |  |

*All ABC transporter sequences were obtained by searching for the Interpro ID corresponding to ABC transporters (IPR003439) at the JGI Nectria website. Additionally, Blastp searches were executed using the known ABC transporters from the most closely related fungus *F. graminearum* and the well-characterized ABC transporters of *S. cerevisiae* as queries. Automated annotation was verified or edited by comparison of amino acid alignments to other ABC transporters.
