# Supplementary material for: The Genome of Nectria haematococca: Contribution of Supernumerary Chromosomes to Gene Expansion
Source: PLoS Genet. 2009 Aug 28;5(8):e1000618. doi: 10.1371/journal.pgen.1000618 (PMC2725324; doi:10.1371/journal.pgen.1000618)
Supplement: Table S5 — Carbohydrate-active enzymes in N. haematococca MPVI compared to other fungi. (0.07 MB DOC) [file pgen.1000618.s010.doc]

| **Fungal species** | **Total GH** | **Total PL** | **GH3** | **GH12** | **GH18** | **GH43** | **GH78** | **GH88** | **GH92** | **GH93** | **GH114** | **PL1** | **PL3** | **PL4** | **CE3** |
| --- | --- | --- | --- | --- | --- | --- | --- | --- | --- | --- | --- | --- | --- | --- | --- |
| *Aspergillus fumigatus Af293* | 263 | 14 | 18 | 4 | 18 | 18 | 6 | 2 | 4 | 3 | 1 | 6 | 3 | 3 | 1 |
| *Aspergillus nidulans FGSC A4* | 261 | 20 | 21 | 1 | 20 | 18 | 9 | 3 | 5 | 2 | 1 | 9 | 5 | 4 | 6 |
| *Aspergillus niger CBS 513.88* | 243 | 8 | 17 | 4 | 14 | 10 | 8 | 1 | 5 | 0 | 2 | 6 | 0 | 2 | 1 |
| *Aspergillus oryzae RIB40* | 284 | 23 | 23 | 4 | 18 | 20 | 8 | 3 | 6 | 3 | 1 | 12 | 3 | 4 | 2 |
| *Cryptococcus neoformans JEC21* | 74 | 3 | 3 | 0 | 4 | 0 | 2 | 0 | 1 | 0 | 1 | 0 | 0 | 1 | 0 |
| *Fusarium graminearum* | 244 | 21 | 22 | 4 | 19 | 17 | 7 | 1 | 0 | 2 | 2 | 9 | 7 | 3 | 5 |
| *Laccaria bicolor* | 163 | 7 | 2 | 3 | 10 | 0 | 0 | 2 | 2 | 0 | 0 | 0 | 0 | 0 | 0 |
| *Magnaporthe oryzae 70-15* | 260 | 4 | 20 | 3 | 15 | 20 | 3 | 1 | 6 | 1 | 0 | 2 | 1 | 1 | 7 |
| ***Nectria haematococca MPVI*** | **329** | **33** | **38** | **6** | **28** | **32** | **11** | **5** | **0** | **4** | **4** | **13** | **11** | **5** | **11** |
| *Neurospora crassa OR74A* | 172 | 4 | 9 | 1 | 12 | 7 | 0 | 0 | 2 | 2 | 1 | 1 | 1 | 1 | 3 |
| *Phanerochaete chrysosporium* | 180 | 4 | 11 | 2 | 11 | 4 | 1 | 1 | 4 | 0 | 0 | 0 | 0 | 0 | 0 |
| *Podospora anserina* | 223 | 7 | 11 | 2 | 20 | 10 | 1 | 0 | 2 | 3 | 1 | 4 | 2 | 1 | 8 |
| *Postia placenta* | 248 | 8 | 9 | 4 | 20 | 1 | 4 | 2 | 6 | 0 | 0 | 0 | 0 | 0 | 0 |
| *Saccharomyces cerevisiae S288C* | 46 | 0 | 0 | 0 | 2 | 0 | 0 | 0 | 0 | 0 | 0 | 0 | 0 | 0 | 0 |
| *Schizosaccharomyces pombe 972h-* | 47 | 0 | 1 | 0 | 1 | 0 | 0 | 0 | 0 | 0 | 0 | 0 | 0 | 0 | 0 |
| *Trichoderma reesei* | 192 | 5 | 13 | 2 | 20 | 2 | 1 | 0 | 7 | 0 | 0 | 0 | 0 | 0 | 2 |
| *Ustilago maydis* | 99 | 1 | 3 | 0 | 3 | 4 | 0 | 0 | 3 | 0 | 0 | 1 | 0 | 0 | 0 |

**Table S5.** Carbohydrate-active enzymes* in *N. haematococca* MPVI compared to other fungi.

*Carbohydrate-active enzymes were detected and annotated using BLAST and HMM-based procedures routinely used for the updates of the CAZy database (http://www.cazy.org/). The total number of glycoside hydrolases (GHs) and polysaccharide lyases (PLs) are shown in the first two columns. Selected families selectively enriched or depleted in *N. haematococca* MPVI are highlighted in red while those with substantially lower abundance appear in blue. The GH12 family contains an extra copy of a putative endoglucanase; Nh48733 is orthologous to FGSG_05851 and Nh122895 is a pseudoparalogous copy.
