# Supplementary material for: The Genome of Nectria haematococca: Contribution of Supernumerary Chromosomes to Gene Expansion
Source: PLoS Genet. 2009 Aug 28;5(8):e1000618. doi: 10.1371/journal.pgen.1000618 (PMC2725324; doi:10.1371/journal.pgen.1000618)
Supplement: Table S6 — The number of cytochrome P450 genes in Nectria haematococca MPVI compared to other fungi. (0.06 MB DOC) [file pgen.1000618.s011.doc]

| **Fungal species** | **Genes*** | **Pseudogenes** | **Total** |
| --- | --- | --- | --- |
|  |  |  |  |
| *Aspergillus fumigatus* | 73 | 1 | 74 |
| *Aspergillus nidulans* | 114 | 8 | 122 |
| *Aspergillus oryzae* | 155 | 9 | 164 |
| *Cryptococcus neoformans* | 5 |  |  |
| *Fusarium graminearum* | 112 |  |  |
| *Magnaporthe oryzae* | 119 | 4 | 123 |
| ***Nectria haematococca MPVI* MPVI** | **150** | **11** | **161** |
| *Neurospora crassa* | 41 |  |  |
| *Phanerochaete chrysosporium* | 149 | 10 | 159 |
| *Saccharomyces cerevisiae* | 3 |  |  |
| *Schizosaccharomyces pombe* | 2 |  |  |
| *Ustilago maydis* | 17 |  |  |
|  |  |  |  |

**Table S6.** The number of cytochrome P450 genes in *Nectria haematococca* MPVI compared to other fungi.

*All cytochrome P450 sequences retrieved from the JGI *N. haematococca* MPVI website (v. 2) by a text search for P450 were placed in a Do-it-yourself Blast server at <http://www.proweb.org/proweb/Tools/WU-blast.html> and compared to a set of about 900 known fungal CYPs. Any new P450s found were named and added to the known set; redundant gene models were removed. After processing, all *N. haematococca* P450s by this method, the new genes were aligned with related genes and examined for indels. In this procedure, intron-exon boundaries were corrected so the sequences would match best to the closest known P450 sequences. After this procedure, a list of CYPs from other *Fusarium* species was examined to find any CYP families that were not in *N. haematococca* MPVI and these were used to search the *N. haematococca* genome by BLAST. The entire process identified 150 CYP genes and 11 pseudogenes in *N. haematococca* MPVI. The Cyp544 family contains two genes in *N. haematococca* MPVI where Nh35331 is orthologous to the *F. graminearum* FGSG_08377 and Nh54370 is the pseudoparalog.
