# Supplementary material for: The Genome of Nectria haematococca: Contribution of Supernumerary Chromosomes to Gene Expansion
Source: PLoS Genet. 2009 Aug 28;5(8):e1000618. doi: 10.1371/journal.pgen.1000618 (PMC2725324; doi:10.1371/journal.pgen.1000618)
Supplement: Table S7 — Number of predicted genes in Nectria haematococca MPVI that contain transcription factor motifs compared to other fungi. (0.10 MB DOC) [file pgen.1000618.s012.doc]

**Table S7.** Number of predicted genes in *Nectria haematococca* MPVI that contain transcription factor motifs compared to other fungi.

|  |  |  | **Fungal species*** | | | |  |
| --- | --- | --- | --- | --- | --- | --- | --- |
| **Superclass** | **Class** | **InterPro**** | ***N. cra.*** | ***M. ory.*** | ***F. gra.*** | ***N. hae.*** | **Conserved***** |
|  |  |  |  |  |  |  |  |
| **β scaffold** |  |  |  |  |  |  |  |
|  | CAAT box | IPR005612 | 1 | 1 | 1 | **1** | 100% |
|  | CBF | IPR001289 | 2 | 2 | 2 | **1** | 100% |
|  | MAD | IPR002100 | 2 | 2 | 2 | **2** | 100% |
|  | HMG | IPR000910 | 10 | 8 | 9 | **7** | 85% |
| **Helix turn-helix** |  |  |  |  |  |  |  |
|  | Heat shock | IPR000232 | 3 | 3 | 3 | **3** | 100% |
|  | Fork head | IPR001766 | 3 | 3 | 3 | **5** | 100% |
|  | Homeobox | IPR001356 | 6 | 4 | 12 | **11** | 81% |
| **Basic domains** |  |  |  |  |  |  |  |
|  | bHLH | IPR001092 | 12 | 7 | 15 | **12** | 83% |
|  | bZIP | IPR004827 | 12 | 11 | 14 | **29** | 55% |
|  | RFX | IPR007668 | 1 | 1 | 1 | **1** | 100% |
| **Zn finger** |  |  |  |  |  |  |  |
|  | GATA | IPR000679 | 6 | 8 | 7 | **8** | 88% |
|  | C2H2 | IPR007087 | 51 | 53 | 64 | **102** | 42% |
|  | CCCH | IPR000571 | 12 | 5 | 17 | **14** | 57% |
| **Binuclear Zn-cluster** |  |  |  |  |  |  |  |
|  | Zn2C6 | IPR001138 | 92 | 54 | 256 | **429** | n.d. |
|  |  | IPR001138 & IPR007219**** | 38 | 54 | 97 | **215** | 35% |
|  |  |  |  |  |  |  |  |

* *N. cra. = Neurospora crassa*; *M. ory.* = *Magnaporthe oryzae*; *F. gra.* = *Fusarium graminearum*; *N. hae.* = *Nectria* *haematococca* MPVI

** InterPro entry for designated motif in the *N. haematococca* JGI database. The related Pfam domain was used in the Broad Institute databases. Matches with a relevance of 0.75 or above are reported.

***Percentage of *N. haematococca* genes in the specified family that display 50% or greater identity to genes in other filamentous fungi.

**** IPR007219 is a fungal specific transcription motif present in a subset of Zn-cluster genes. The separate listing for proteins containing both motifs provides a more conservative, but robust, estimate of the number of binuclear Zn-cluster genes.

The transcription factor Nh92541 is orthologous to *F. graminearum* FGSG_05158 while Nh82690 is a pseudoparalog.
