# Supplementary material for: The Genome of Nectria haematococca: Contribution of Supernumerary Chromosomes to Gene Expansion
Source: PLoS Genet. 2009 Aug 28;5(8):e1000618. doi: 10.1371/journal.pgen.1000618 (PMC2725324; doi:10.1371/journal.pgen.1000618)
Supplement: Table S8 — The number of chromatin genes in N. haematococca MPVI compared to other fungi. (0.05 MB DOC) [file pgen.1000618.s013.doc]

**Table S8.** The number of chromatin genes* in *N. haematococca* MPVI compared to other fungi.

| **Protein Class** | ***N. hae.*** | ***F. gra.*** | ***M. ory.*** | ***N. cra.*** | ***S. cer.*** | ***S. pom.*** |
| --- | --- | --- | --- | --- | --- | --- |
|  |  |  |  |  |  |  |
| Histones and Histone Linker Proteins | **10** | 9 | 10 | 8 | 11 | 11 |
| Nucleosome Organization: Assembly and Displacement | **75** | 70 | 66 | 69 | 65 | 68 |
| Histone Modifications | **53** | 49 | 58 | 48 | 41 | 38 |
| Histone Modification-Associated Proteins and Complexes | **10** | 9 | 10 | 10 | 11 | 12 |
| Modified-Histone Binding Proteins | **9** | 9 | 7 | 9 | 11 | 12 |
| DNA Modifying Proteins | **2** | 2 | 2 | 2 | 0 | 1 |
| RNAi Components | **7** | 9 | 7 | 7 | 0 | 5 |
| Condensin Complex Components | **7** | 7 | 7 | 7 | 7 | 7 |
| Totals | **173** | 164 | 167 | 160 | 146 | 154 |
|  |  |  |  |  |  |  |

**N. crassa* chromatin-associated and RNAi-associated proteins displayed at The Chromatin Database (www.chromdb.org) were used as queries to search Joint Genome Institute catalog proteins for *N. haematococca* MPVI to identify putative homologs. Blastp e-values were set initially at e-50 and dropped down to e-10 for successive blastp searches to identify divergent proteins. Preliminary evolutionary assignments were made using a specialized local ChromDB BLAST program.

*N. hae.* = *N. haematococca* MPVI, *F. gra.* = *F. graminearum*, *M. ory* = *M. oryzae, N. cra.* = *N. crassa, S. cer*. = *S*. *cerevisiae,*

*S. pom.* = *S. pombe*
