# Supplementary material for: The Genome of Nectria haematococca: Contribution of Supernumerary Chromosomes to Gene Expansion
Source: PLoS Genet. 2009 Aug 28;5(8):e1000618. doi: 10.1371/journal.pgen.1000618 (PMC2725324; doi:10.1371/journal.pgen.1000618)
Supplement: Table S9 — Distribution of repeat elements in the genome of Nectria haematococca MPVI. (0.08 MB DOC) [file pgen.1000618.s014.doc]

**Table S9.** Distribution of repeat elements in the genome of *Nectria haematococca* MPVI

| **Bulk DNA** | **Repeated DNA Class*** | **Total**** | **Mapped** | **Chr. 1 to 13***** | **Chr. 14 to 17** | **Unmapped****** |
| --- | --- | --- | --- | --- | --- | --- |
|  |  |  |  |  |  |  |
| **Unique** |  |  |  |  |  |  |
| bp |  | 48,661,382 | 46,175,441 | 44,494,785 | 1,680,656 | 2,485,941 |
| % of genome |  | 94.9% | 90.0% | 86.8% | 3.3% | 4.9% |
| **Repeated*** |  |  |  |  |  |  |
| bp |  | 2,625,115 | 1,152,618 | 780,551 | 372,067 | 1472,497 |
| % of genome |  | 5.1% | 2.2% | 1.5% | 0.7% | 2.9% |
| % of repeated |  | 100% | 43.9% | 29.7% | 14.2% | 56.1% |
|  | **TE class I** |  |  |  |  |  |
|  | bp | 1,080,615 | 492,637 | 395,715 | 96,922 | 587,978 |
|  | % of repeated | 41% | 19% | 15% | 4% | 22% |
|  | **TE class II** |  |  |  |  |  |
|  | bp | 646,734 | 416,423 | 240,292 | 176,131 | 230,311 |
|  | % of repeated | 25% | 16% | 9% | 7% | 9% |
|  | **Duplication** |  |  |  |  |  |
|  | bp | 390,206 | 107,786 | 37,221 | 70,565 | 282,420 |
|  | % of repeated | 15% | 4% | 1% | 3% | 11% |
|  | **Unclassified** |  |  |  |  |  |
|  | bp | 257,318 | 108,434 | 95,383 | 13,051 | 148,884 |
|  | % of repeated | 10% | 4% | 4% | <1% | 6% |
|  | **Mixed** |  |  |  |  |  |
|  | bp | 250,242 | 27,338 | 10,421 | 16,917 | 222,904 |
|  | % of repeated | 10% | 1% | <1% | <1% | 8% |
|  |  |  |  |  |  |  |

***** Repeated elements were detected by the *de novo* repeat finding software “RepeatScout” and grouped into 55 repeat families, each of

which was characterized by a “consensus” repeat element generated by the software. Some families were split further to generate a

total of 84 repeat families. Consensus elements were used as baits in BLASTx searches against the non-redundant GenBank database as well as all publicly available fungal genomes to identify active or mutated transposable elements. tBLASTn searches against the *N. haematococca* genome were carried out to identify active or mutated homologues of putative transposable elements previously identified in other organisms. The “Mixed” class of repeats is made up of roughly equal amounts of Class I, Class II and presumed gene duplications.

** Percentages are based on a total haploid genome of 51,286,497 bp. Percentages in bold indicate the fraction in each repeat class based on 2,625,115 bp of repeated DNA.

*** Chromosomes 1 to 13 are predicted to be normal chromosomes, while chromosomes 14. 15, and 17 are supernumerary chromosomes. Chromosomes 1-13 contain 45,275,336 bp or 96.3% of the mapped DNA. Chromosomes 14-17 contain 2,052,723 bp or 4.5% of the mapped

DNA.

**** Unmapped regions are comprised of 136 contigs of ~29 kbp average length (compared to ~76 to 2,130 kbp for the 72 mapped contigs). Overall, unmapped contigs are more AT-rich than mapped contigs and may contain a significant amount of centromeric or pericentric DNA.
