# Supplementary material for: The Genome of Nectria haematococca: Contribution of Supernumerary Chromosomes to Gene Expansion
Source: PLoS Genet. 2009 Aug 28;5(8):e1000618. doi: 10.1371/journal.pgen.1000618 (PMC2725324; doi:10.1371/journal.pgen.1000618)
Supplement: Table S10 — Properties of the chromosomes and genes on each chromosome in N. haematococca MPVI. (0.09 MB DOC) [file pgen.1000618.s015.doc]

**Table S10.** Properties of the chromosomes and genes on each chromosome in *N. haematococca* MPVI.

| **Chromosome** |  | | **Chromosome property** | | | | | | | | | |  | | **Gene property** | | | |
| --- | --- | --- | --- | --- | --- | --- | --- | --- | --- | --- | --- | --- | --- | --- | --- | --- | --- | --- |
|  |  | | **Assembled size (bp)** | | **Optical map size (Mb)** | | **% GC*** | | **Number of genes** | | **Density (genes/Mb)** | |  | | **Length (nt)** | | **Exons/ gene** | |
|  |  | |  | |  | |  | |  | |  | |  | |  | |  | |
| 1 | |  | | 6,369,736 | | 6.52 | | 52.9 | | 1840 | | 289 | |  | | 1825 | | 3.0 |
| 2 | |  | | 4,350,924 | | 5.57 | | 52.5 | | 1359 | | 312 | |  | | 1752 | | 3.0 |
| 3 | |  | | 4,591,189 | | 5.30 | | 51.9 | | 1418 | | 309 | |  | | 1731 | | 3.0 |
| 4 | |  | | 4,237,857 | | 4.41 | | 52.3 | | 1221 | | 279 | |  | | 1825 | | 3.0 |
| 5 | |  | | 3,973,246 | | 4.02 | | 52.2 | | 1223 | | 308 | |  | | 1659 | | 3.0 |
| 6 | |  | | 3,621,839 | | 3.92 | | 51.5 | | 1218 | | 336 | |  | | 1636 | | 3.0 |
| 7 | |  | | 2,948,412 | | 3.83 | | 49.7 | | 1062 | | 360 | |  | | 1549 | | 3.2 |
| 8 | |  | | 3,391,797 | | 3.73 | | 52.0 | | 1093 | | 322 | |  | | 1748 | | 3.1 |
| 9 | |  | | 3,013,008 | | 3.22 | | 52.0 | | 994 | | 330 | |  | | 1737 | | 3.1 |
| 10 | |  | | 2,731,896 | | 3.00 | | 51.4 | | 952 | | 349 | |  | | 1661 | | 3.1 |
| 11 | |  | | 2,284,780 | | 2.72 | | 49.5 | | 832 | | 364 | |  | | 1557 | | 3.1 |
| 12 | |  | | 2,235,368 | | 2.59 | | 50.0 | | 870 | | 374 | |  | | 1530 | | 3.2 |
| 13 | |  | | 1,298,360 | | 2.19 | | 49.5 | | 430 | | 331 | |  | | 1565 | | 3.3 |
| 14 | |  | | 1,260,466 | | 1.57 | | 48.7 | | 290 | | 230 | |  | | 1376 | | 2.9 |
| 15 | |  | | 346,570 | | 0.75 | | 46.2 | | 71 | | 205 | |  | | 1327 | | 2.9 |
| 16 | |  | | 215,166 | | 0.56 | | 49.2 | | 75 | | 349 | |  | | 1554 | | 3.2 |
| 17 | |  | | 230,523 | | 0.53 | | 48.4 | | 57 | | 247 | |  | | 1484 | | 3.1 |
| unmapped | |  | | 3,958,438 | |  | |  | | 702 | | 187 | |  | | 1427 | | 3.3 |
|  | |  | |  | |  | |  | |  | |  | |  | |  | |  |

The total assembled genome size is 51,149,575 bp and the size by optical map is 54.43 Mb.

*The supernumerary chromosomes (14, 15 and 17) have a significantly lower GC content (48.2%) than the other chromosomes (51.7%) as shown by the Mann-Whitney test (p=0.008073
