# Supplementary material for: The Genome of Nectria haematococca: Contribution of Supernumerary Chromosomes to Gene Expansion
Source: PLoS Genet. 2009 Aug 28;5(8):e1000618. doi: 10.1371/journal.pgen.1000618 (PMC2725324; doi:10.1371/journal.pgen.1000618)
Supplement: Table S11 — The protein kinases of N. haematococca MPVI compared to S. cerevisiae. (0.03 MB DOC) [file pgen.1000618.s016.doc]

**Table S11.** The protein kinases of *N. haematococca* MPVI compared to *S. cerevisiae.*

| **Protein kinase class** | ***N. haematococca* MPVI** | ***S. cerevisiae*** |
| --- | --- | --- |
|  |  |  |
| STE | 12 | 14 |
| CMGC | 19 | 11 |
| CK1 | 3 | 4 |
| CAMK | 15 | 21 |
| AGC | 13 | 17 |
| HK | 1 | 1 |
| Other | 21 | 38 |
|  |  |  |

All *N. haematococca* MPVI genes were compared to a set of 99 well-documented kinase catalytic domains from diverse kinase families. Sequences with significant matches to multiple known kinase genes were assigned to families by clustering them together with known kinases from human, *Caenorhabditis elegans, Drosphila melanogaster,* and *S. cerevisiae*. Briefly, protein sequences were compared in an all-against-all blast search with a maximum E-value cutoff of 10.0. Sequences were hierarchically clustered using a maximum linkage approach, and assigned to families based on their positions within clades of known kinase families.
