# Supplementary material for: The Genome of Nectria haematococca: Contribution of Supernumerary Chromosomes to Gene Expansion
Source: PLoS Genet. 2009 Aug 28;5(8):e1000618. doi: 10.1371/journal.pgen.1000618 (PMC2725324; doi:10.1371/journal.pgen.1000618)
Supplement: Table S12 — The number of polyketide synthases (PKS) and nonribosomal peptide synthetases (NRPS) of Nectria haematococca MPVI compared to other fungi. (0.06 MB DOC) [file pgen.1000618.s017.doc]

**Table S12**. The number of polyketide synthases (PKS) and nonribosomal peptide synthetases (NRPS) of *Nectria haematococca* MPVI compared to other fungi

| **Fungal species** | **PKS** | **NRPS** | **PKS-NRPS** | **Total** |
| --- | --- | --- | --- | --- |
|  |  |  | **NRPS-PKS** |  |
|  |  |  |  |  |
| *Aspergillus oryzae* | 26 | 14 | 4 | 44 |
| *Aspergillus nidulans* | 26 | 13 | 1 | 40 |
| *Aspergillus fumigatus* | 13 | 13 | 1 | 27 |
| *Neurospora crassa* | 7 | 3 | 0 | 10 |
| *Magnaporthe oryzae* | 20 | 6 | 8 | 34 |
| *Gibberella moniliformis* | 12 | 16 | 3 | 31 |
| *Fusarium graminearum* | 14 | 19 | 1 | 34 |
| ***Nectria haematococca* MPVI** | **12** | **12** | **1** | **25** |
| *Botryotinia fuckeliana* | 17 | 10 | 2 | 29 |
| *Cochliobolus heterostrophus* | 23 | 11 | 2 | 36 |
|  |  |  |  |  |

All candidate genes from the genomic sequence were retrieved with BLASTp, using representative fungal PKS and NRPS amino acid sequences as queries. The automatically annotated candidate genes were retrieved from the *N. haematococca* MPVI JGI website (version 2).  In order to manually verify the annotation, each predicted ORF was used as a BLASTp query to retrieve the most closely related genes from GenBank. The predicted ORF was aligned with the top BLASTp hits, and a gene genealogy was generated by neighbor-joining and parsimony. The gene genealogy was used to estimate which *N. haematococca* MPVI genes have orthologs in other fungi and to make predictions of gene function.  Corrections to the automated annotation were made by reconciling each ORF to the amino acid sequence of its orthologs, if they were found, or to the most closely related paralogs, if no orthologs were found.  Discrepancies in 5' and 3' boundaries and intron predictions were corrected by retrieving the nucleotide sequence and manually predicting exon boundaries using the amino acid alignment and predicted intron splice site motifs as a guide.
