# Supplementary material for: The Genome of Nectria haematococca: Contribution of Supernumerary Chromosomes to Gene Expansion
Source: PLoS Genet. 2009 Aug 28;5(8):e1000618. doi: 10.1371/journal.pgen.1000618 (PMC2725324; doi:10.1371/journal.pgen.1000618)
Supplement: Table S13 — Distribution of Small Secreted Proteins (SSP) among filamentous Ascomycetes as identified by SignalP. (0.05 MB DOC) [file pgen.1000618.s018.doc]

**Table S13.** Distribution of Small Secreted Proteins (SSP) among filamentous Ascomycetes as identified by SignalP.

| **Fungal Species** | **SSP*** | **%SSP**** |
| --- | --- | --- |
|  |  |  |
| *Aspergillus flavus* | 713 | 5.66 |
| *Botrytis cinerea* | 926 | 5.63 |
| *Fusarium graminearum* | 732 | 5.49 |
| *Fusarium oxysporum* | 974 | 5.49 |
| *Fusarium verticillioides* | 780 | 5.50 |
| *Magnaporthe oryzae* | 1146 | 8.92 |
| *Mycosphaerella fijiensis* | 479 | 4.64 |
| *Mycosphaerella graminicola* | 687 | 6.03 |
| ***Nectria hematococca*** | **746** | **4.75** |
| *Neurospora crassa* | 479 | 4.87 |
| *Sclerotinia sclerotiorum* | 769 | 5.30 |
| *Stagonospora nodorum* | 1081 | 6.51 |
| *Trichoderma virens* | 528 | 4.53 |
|  |  |  |

*Eukaryotic profile settings were used for this analysis. A protein was designated as a secreted protein if its SignalP d-score met threshold [102]. Small secreted proteins were defined as being <300bp.

**Small secreted proteins calculated as a percentage of the total annotated proteins for that fungus.
